# Supplementary material for: Multiomics analysis identifies novel facilitators of human dopaminergic neuron differentiation
Source: EMBO Rep. 2023 Dec 19;25(1):17. doi: 10.1038/s44319-023-00024-2 (PMC10897179; doi:10.1038/s44319-023-00024-2)

Appendix data

Multiomics analysis identifies novel facilitators of human dopaminergic neuron differentiation

Table of Contents

Appendix Figure Legends.…...…………………………………………...………2
Appendix Figure S1……………………………………………………….....3
Appendix Figure S2……………………………………………………….....4
Appendix Figure S3……………………………………………………….....5
Appendix Figure S4……………………………………………………….....6

Appendix Figure Legends

Appendix Figure S1. A) Live imaging for early and late transductions with shRNA lentiviral particles across time. Bar plots representing GFP+ cells at day 15 of analysis **B)** Live imaging for early and late transduction with lentiviral particles for the overexpression of LBX1, NHLH1 and NR2F2. Bar plots representing GFP+ cells at day 15 of analysis. Scale bar = 200 µm.

Appendix Figure S2. A) Images of high-content imaging analysis from Figure 4 and Figure EV4 with signal masks for both TH and MAP2 stainings included. Late transduction with indicated shRNA particles in mDANs derived from **(A)** 17608 cells, **(B)** K2135 cells, **(C)** TH-Rep1 cells, and **(D)** TH-Rep2 cells. Scale bar = 100 µm.

Appendix Figure S3. A) Loci and expression of the three genes coding for miR-124 **B)** Live imaging of cells under ABX464 treatment and NHLH1 KD at day 14 of differentiation **C)** Flow cytometry analysis showing the differences in shape between neurons treated with ABX464 and non-treated **D)** Expression Ct values detected by TaqMan and RT-qPCR for miR-1243p and NHLH1, respectively, in samples treated with ABX464 and under NHLH1 KD.

Appendix Figure S4. Live imaging of the GFP levels as a read-out of transduction efficiency in cells under GW3965 and Rapamycin treatment and LBX1 KD at day 14 of differentiation. Scale bar = 200 µm.

Appendix Figure S1

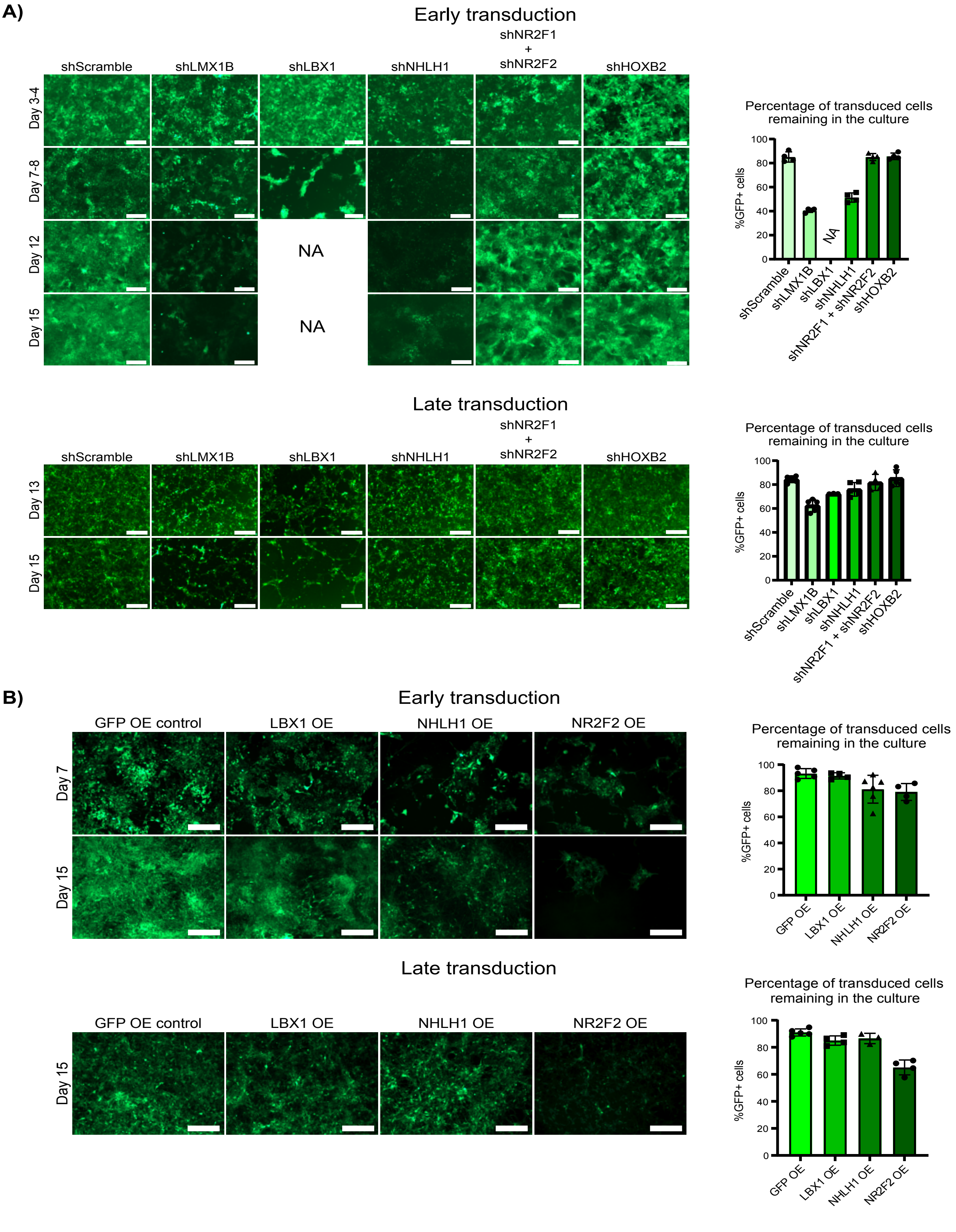

Appendix Figure S2

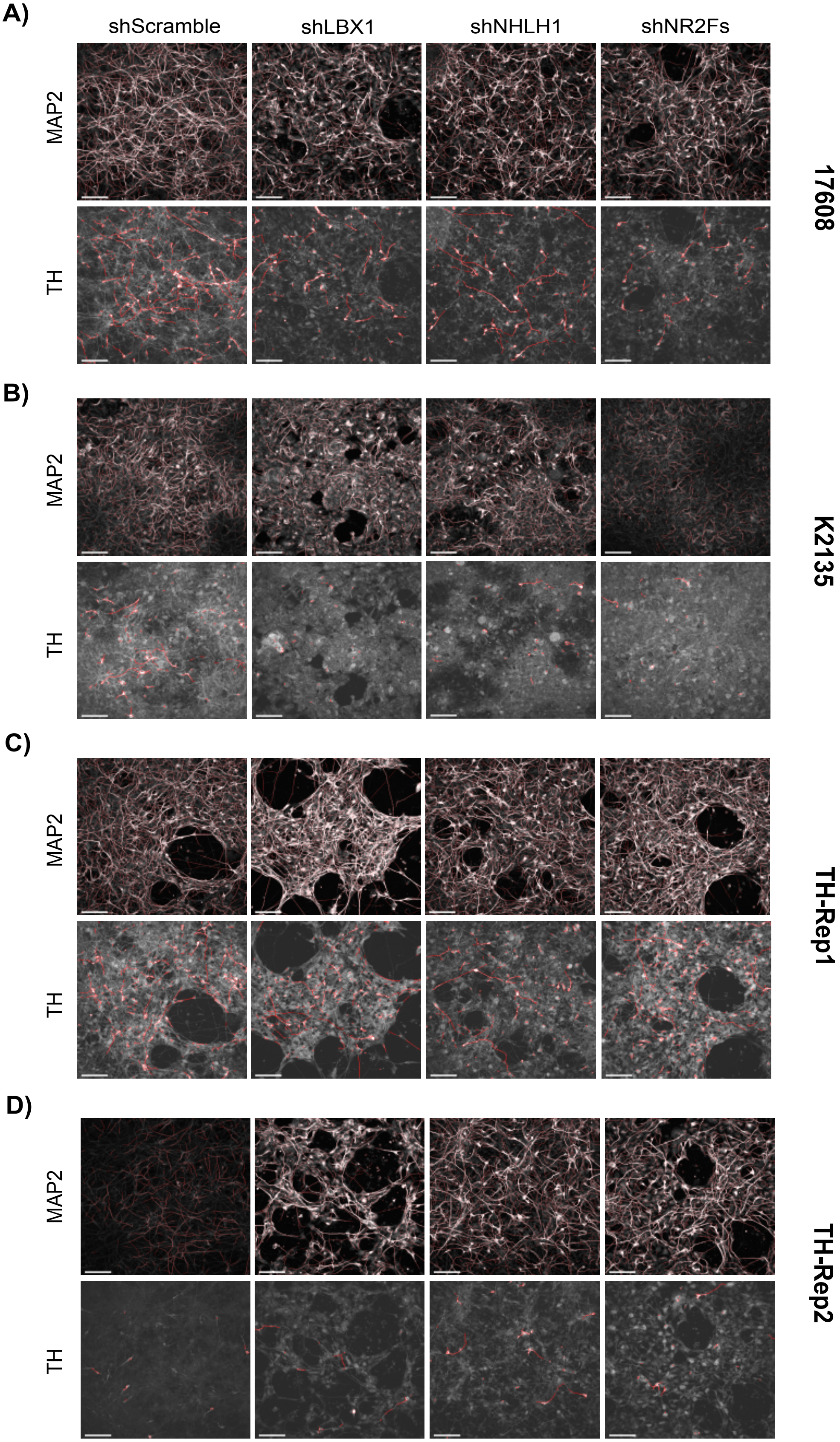

Appendix Figure S3

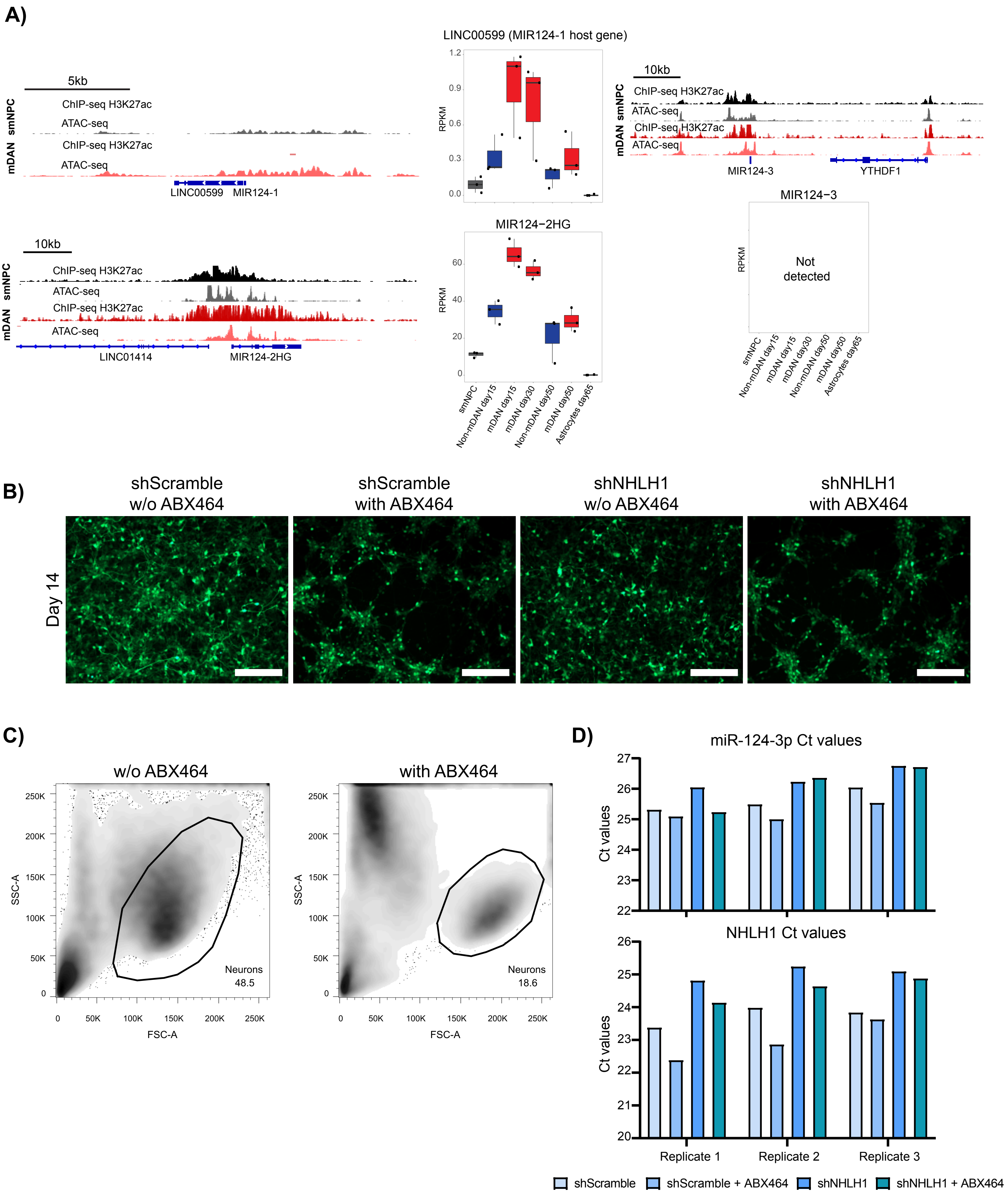

Appendix Figure S4

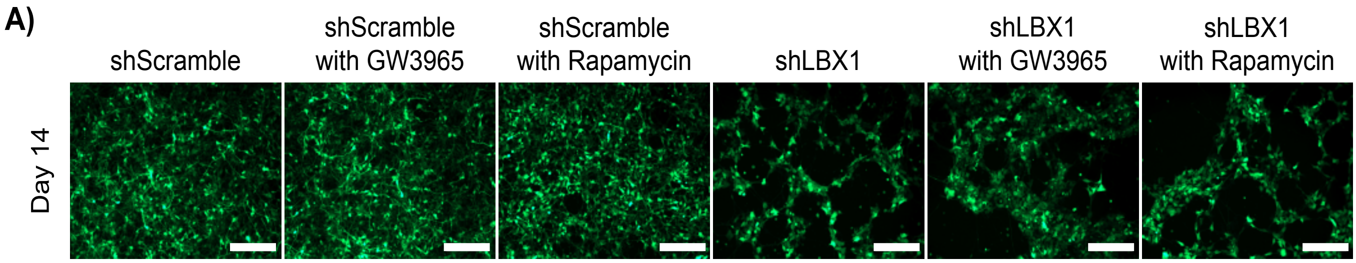

Supplement: Supplementary file 1 — Appendix Figures and Legends [file 44319_2023_24_MOESM1_ESM.pdf]
