# Supplementary figures and images for: Multiomics analysis identifies novel facilitators of human dopaminergic neuron differentiation
Source: EMBO Rep. 2023 Dec 19;25(1):17. doi: 10.1038/s44319-023-00024-2 (PMC10897179; doi:10.1038/s44319-023-00024-2)

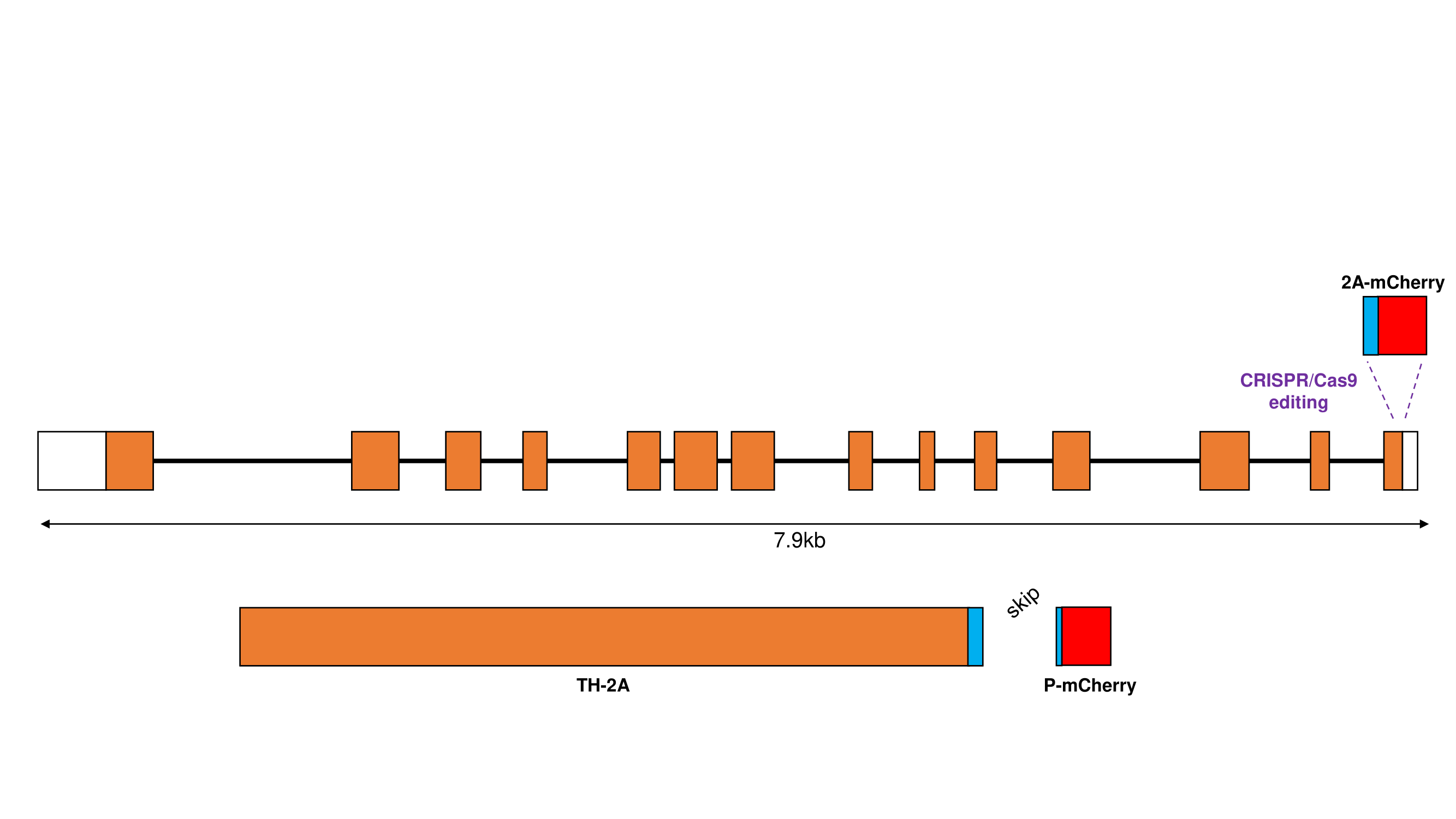

Supplement: Supplementary file 7 — Source Data Fig. 1 [file 44319_2023_24_MOESM7_ESM.zip › Figure 1/1A/mCherry_construct.tiff]

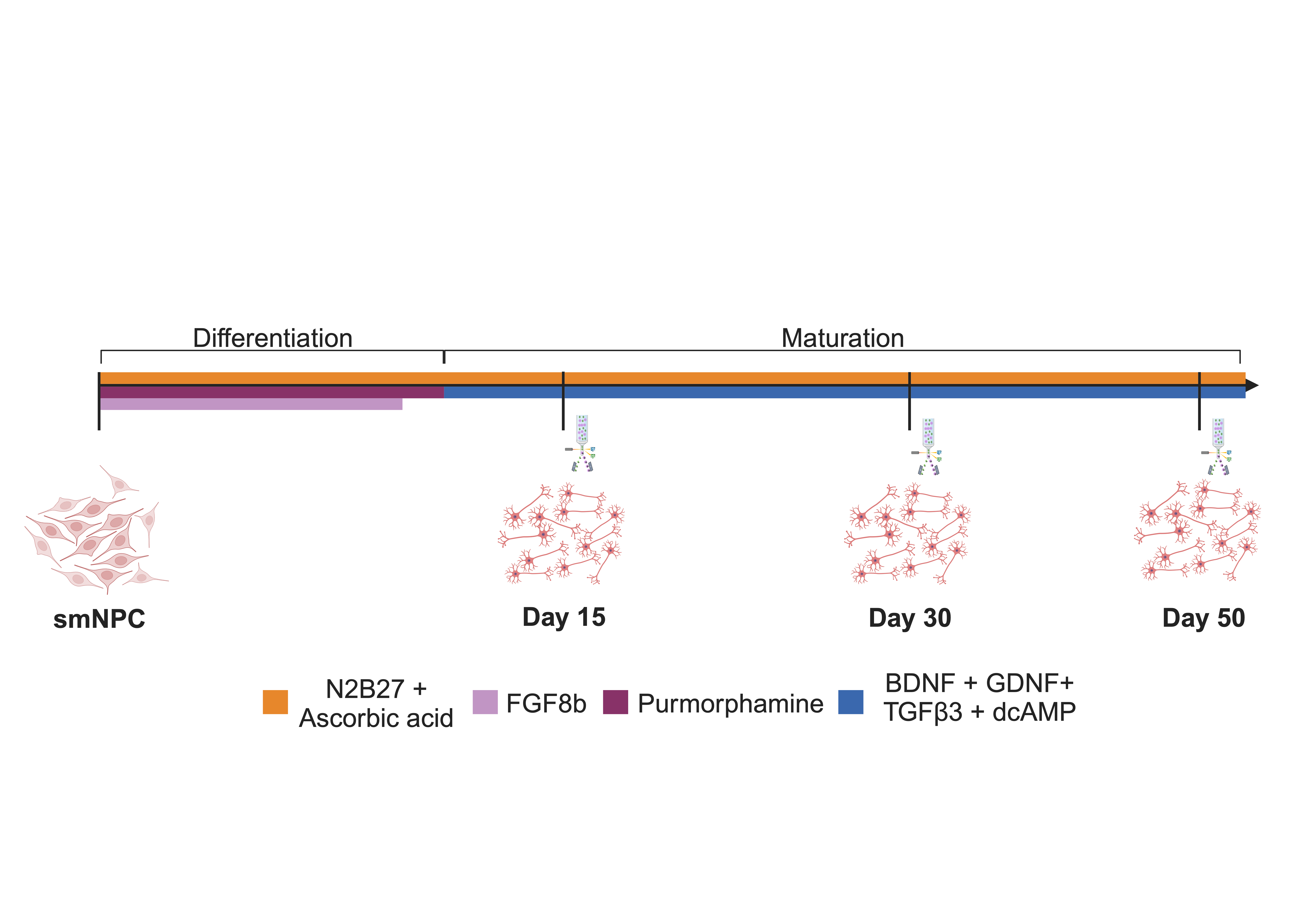

Supplement: Supplementary file 7 — Source Data Fig. 1 [file 44319_2023_24_MOESM7_ESM.zip › Figure 1/1A/DA_differentiation_protocol.tif]

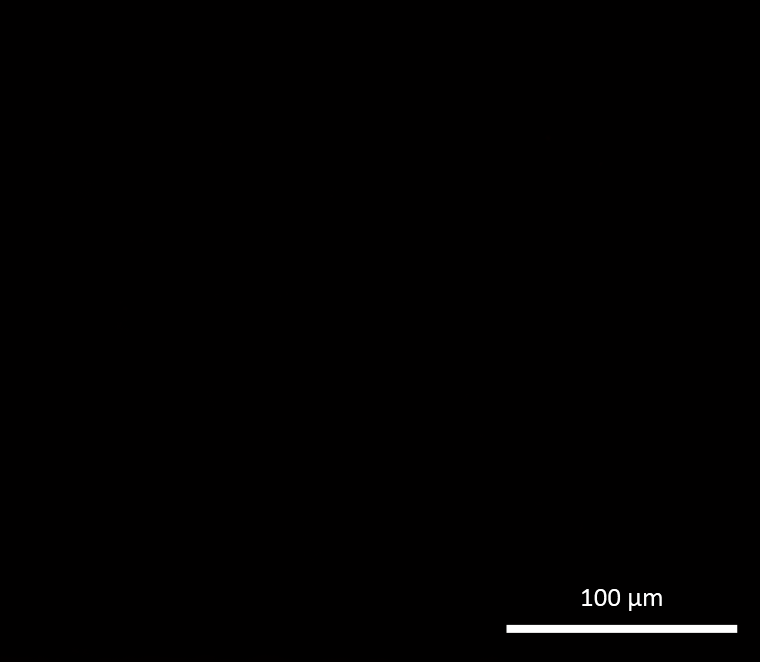

Supplement: Supplementary file 7 — Source Data Fig. 1 [file 44319_2023_24_MOESM7_ESM.zip › Figure 1/1B/HFFTHmCherry_LiveImaging_-sort-03-Image Export-11/HFFTHmCherry_LiveImaging_-sort-03-Image Export-11_mCherry_2.tif]

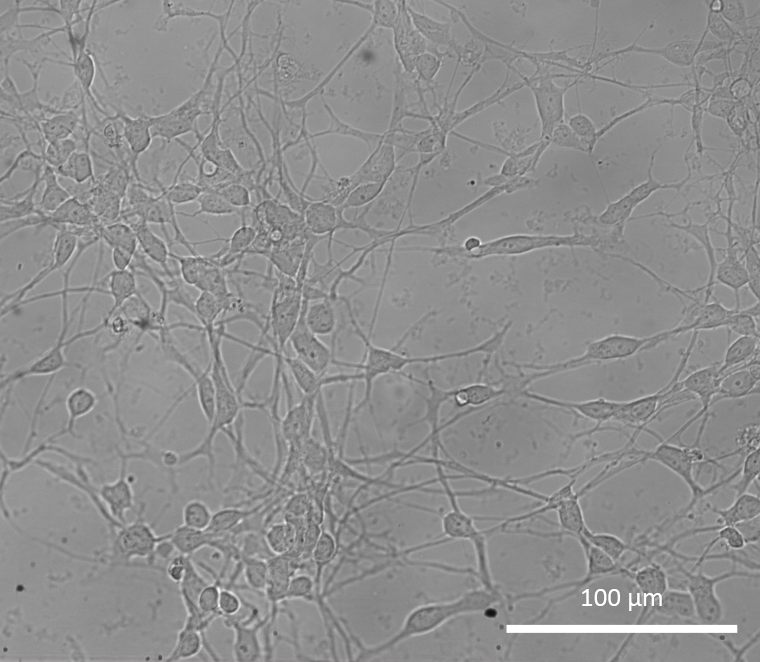

Supplement: Supplementary file 7 — Source Data Fig. 1 [file 44319_2023_24_MOESM7_ESM.zip › Figure 1/1B/HFFTHmCherry_LiveImaging_-sort-03-Image Export-11/HFFTHmCherry_LiveImaging_-sort-03-Image Export-11_c1-2.tif]

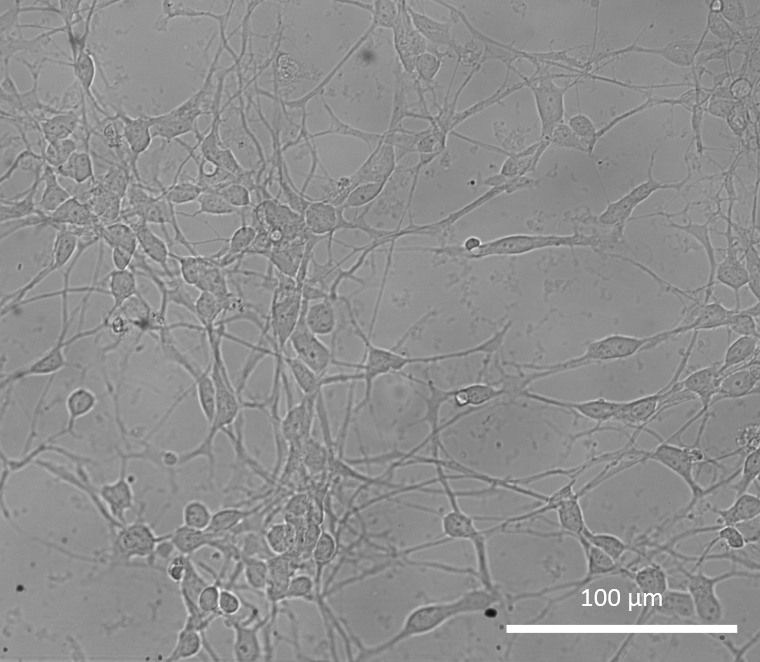

Supplement: Supplementary file 7 — Source Data Fig. 1 [file 44319_2023_24_MOESM7_ESM.zip › Figure 1/1B/HFFTHmCherry_LiveImaging_-sort-03-Image Export-11/HFFTHmCherry_LiveImaging_-sort-03-Image Export-11_TL Phase_1.tif]

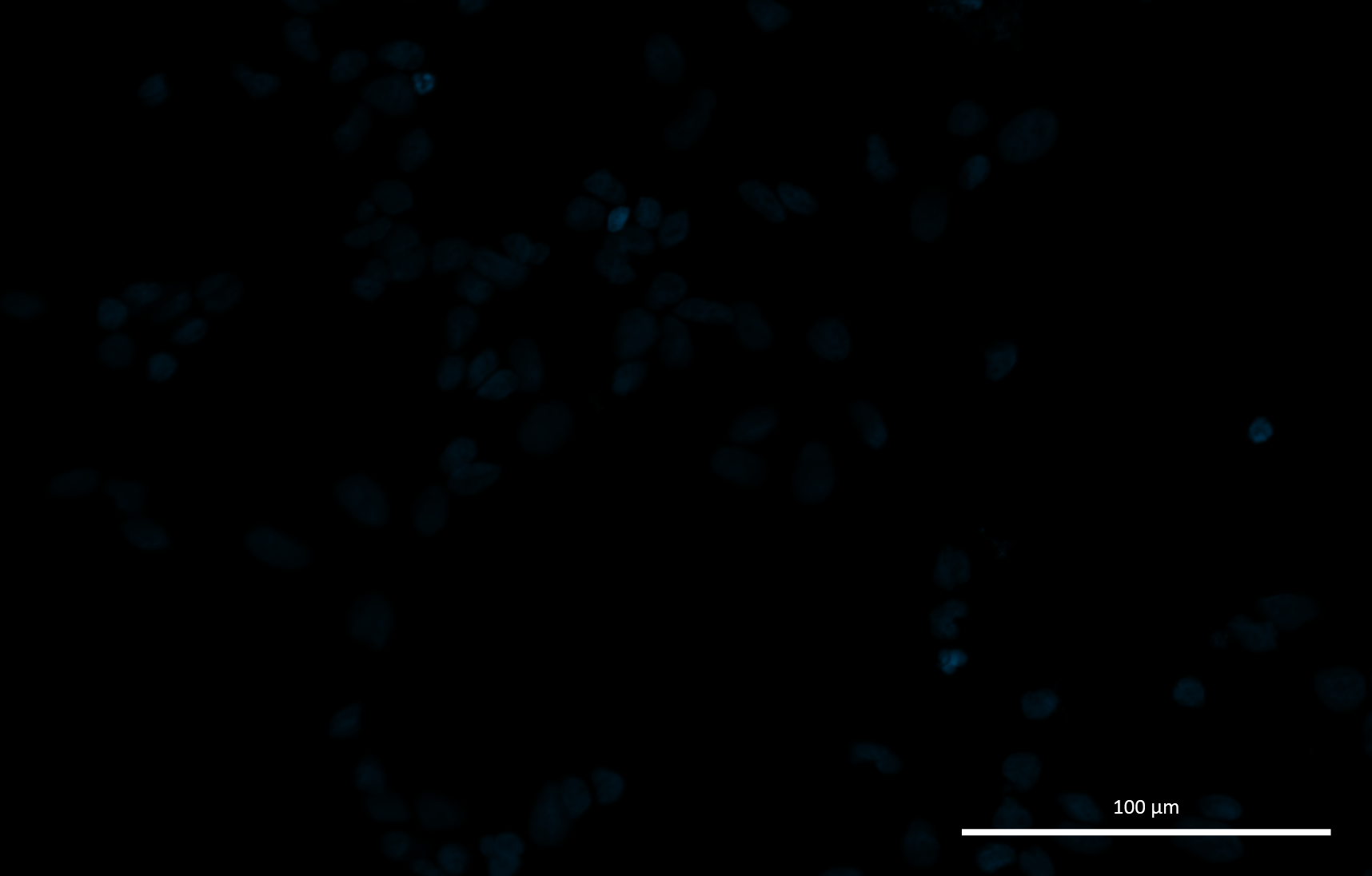

Supplement: Supplementary file 7 — Source Data Fig. 1 [file 44319_2023_24_MOESM7_ESM.zip › Figure 1/1B/HFFTH_D22_TH647_NeuN488_unsorted-03-Image Export-05/HFFTH_D22_TH647_NeuN488_unsorted-03-Image Export-05_DAPI_3.tif]

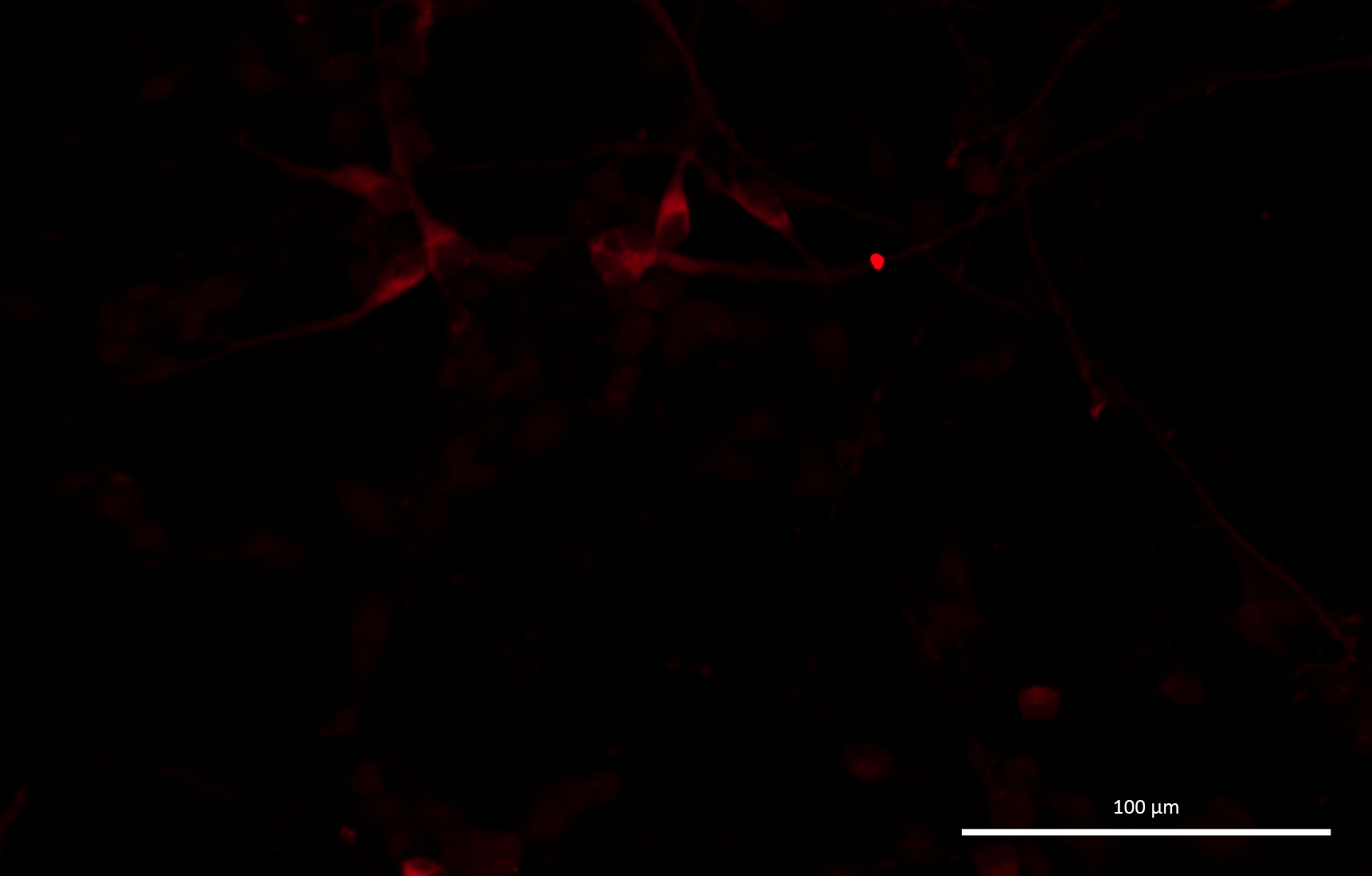

Supplement: Supplementary file 7 — Source Data Fig. 1 [file 44319_2023_24_MOESM7_ESM.zip › Figure 1/1B/HFFTH_D22_TH647_NeuN488_unsorted-03-Image Export-05/HFFTH_D22_TH647_NeuN488_unsorted-03-Image Export-05_Alexa Fluor 647_1.tif]

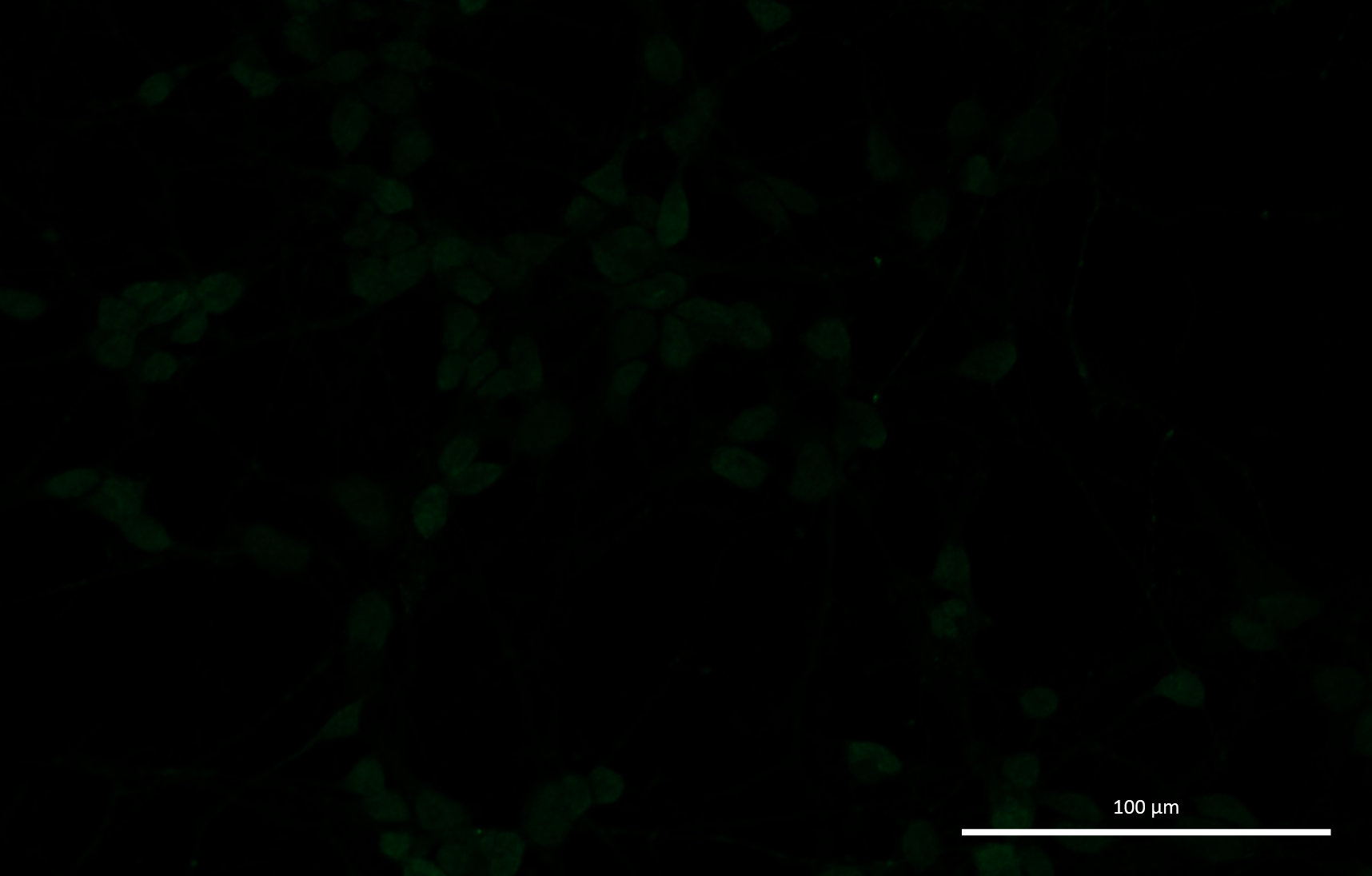

Supplement: Supplementary file 7 — Source Data Fig. 1 [file 44319_2023_24_MOESM7_ESM.zip › Figure 1/1B/HFFTH_D22_TH647_NeuN488_unsorted-03-Image Export-05/HFFTH_D22_TH647_NeuN488_unsorted-03-Image Export-05_Alexa Fluor 488_2.tif]

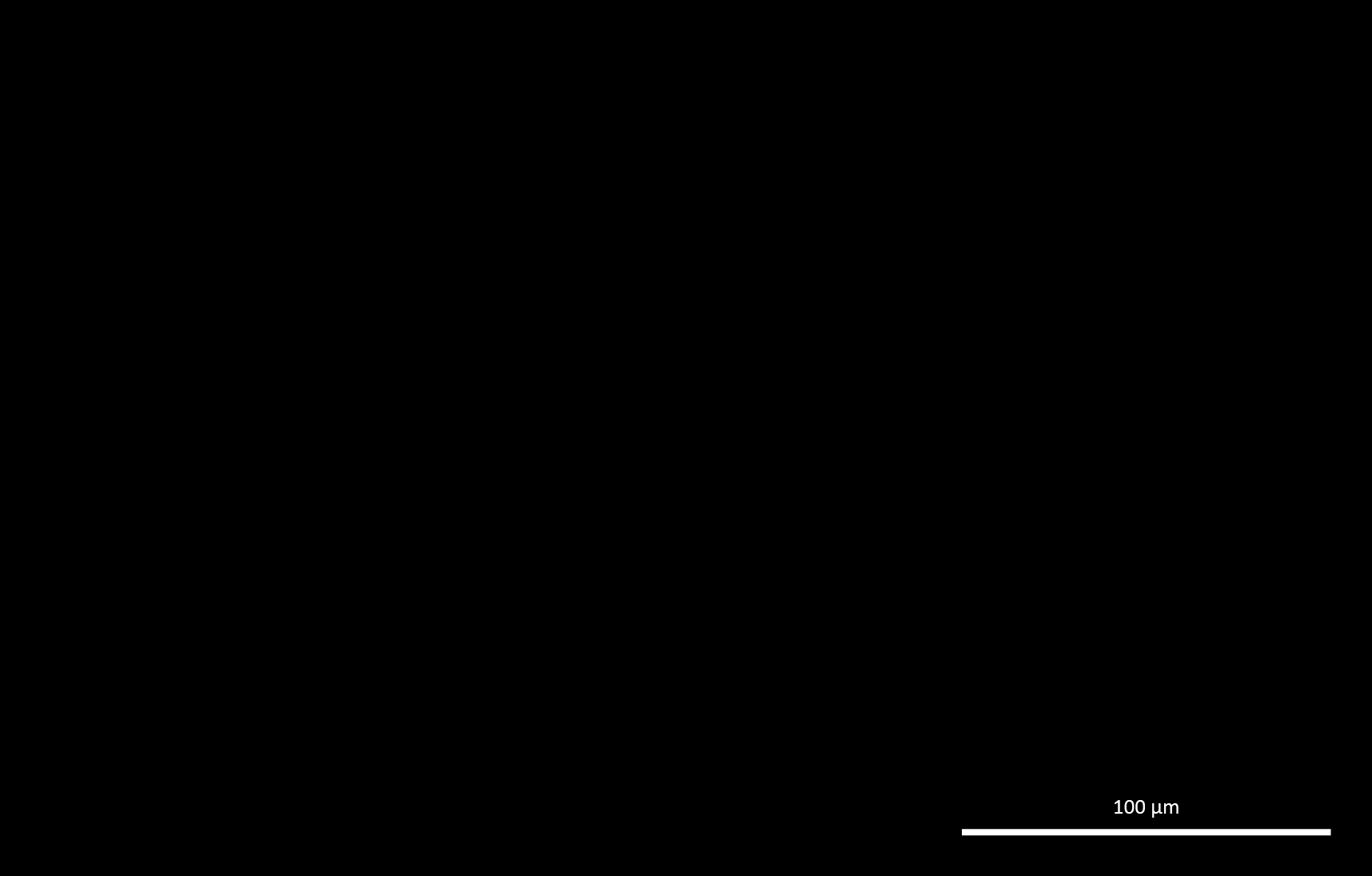

Supplement: Supplementary file 7 — Source Data Fig. 1 [file 44319_2023_24_MOESM7_ESM.zip › Figure 1/1B/HFFTH_D22_NegControl_-Image Export-04/HFFTH_D22_-Control_647-02-Image Export-04_Alexa Fluor 647_1.tif]

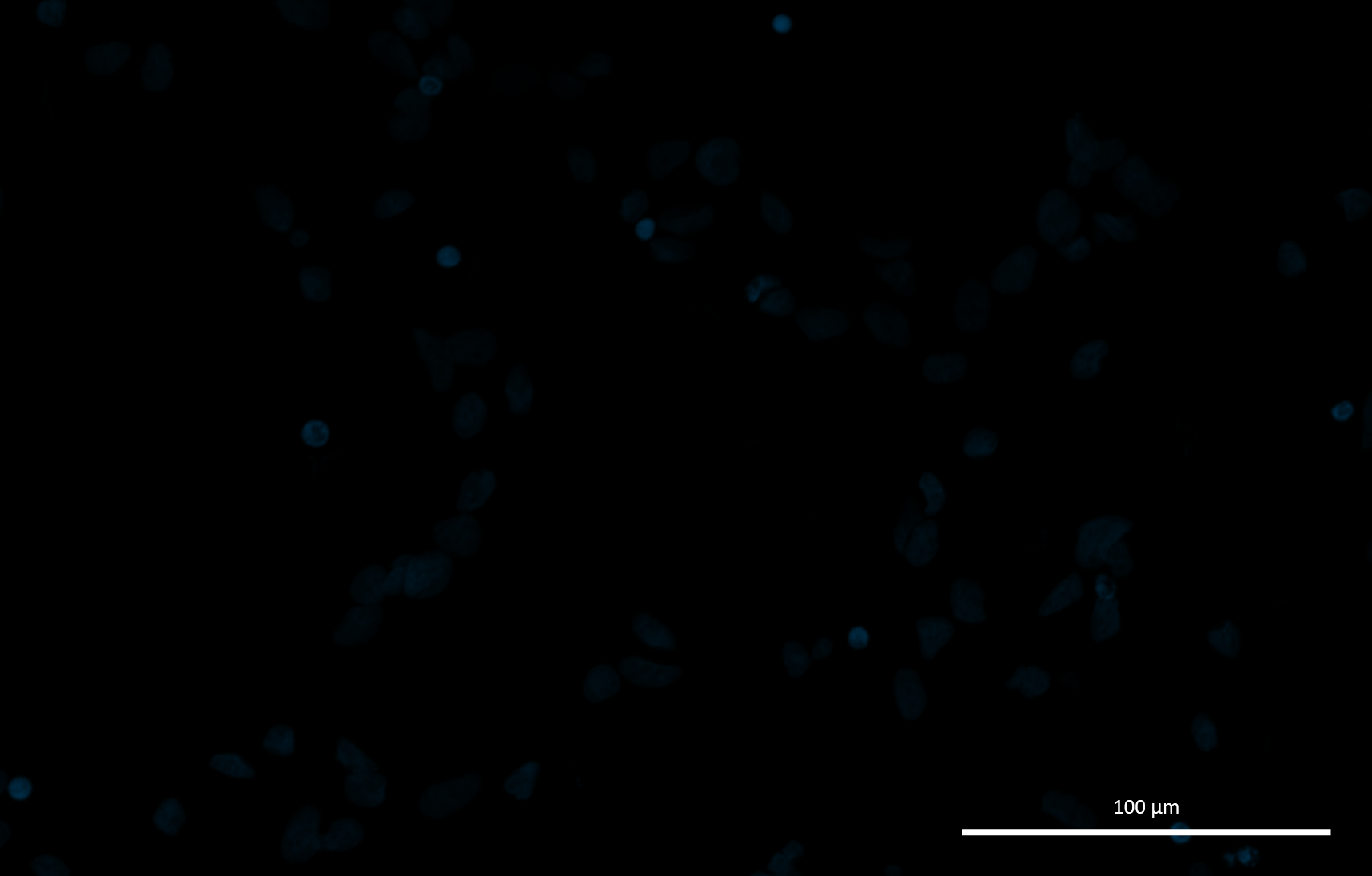

Supplement: Supplementary file 7 — Source Data Fig. 1 [file 44319_2023_24_MOESM7_ESM.zip › Figure 1/1B/HFFTH_D22_NegControl_-Image Export-04/HFFTH_D22_-Control_647-02-Image Export-04_DAPI_3.tif]

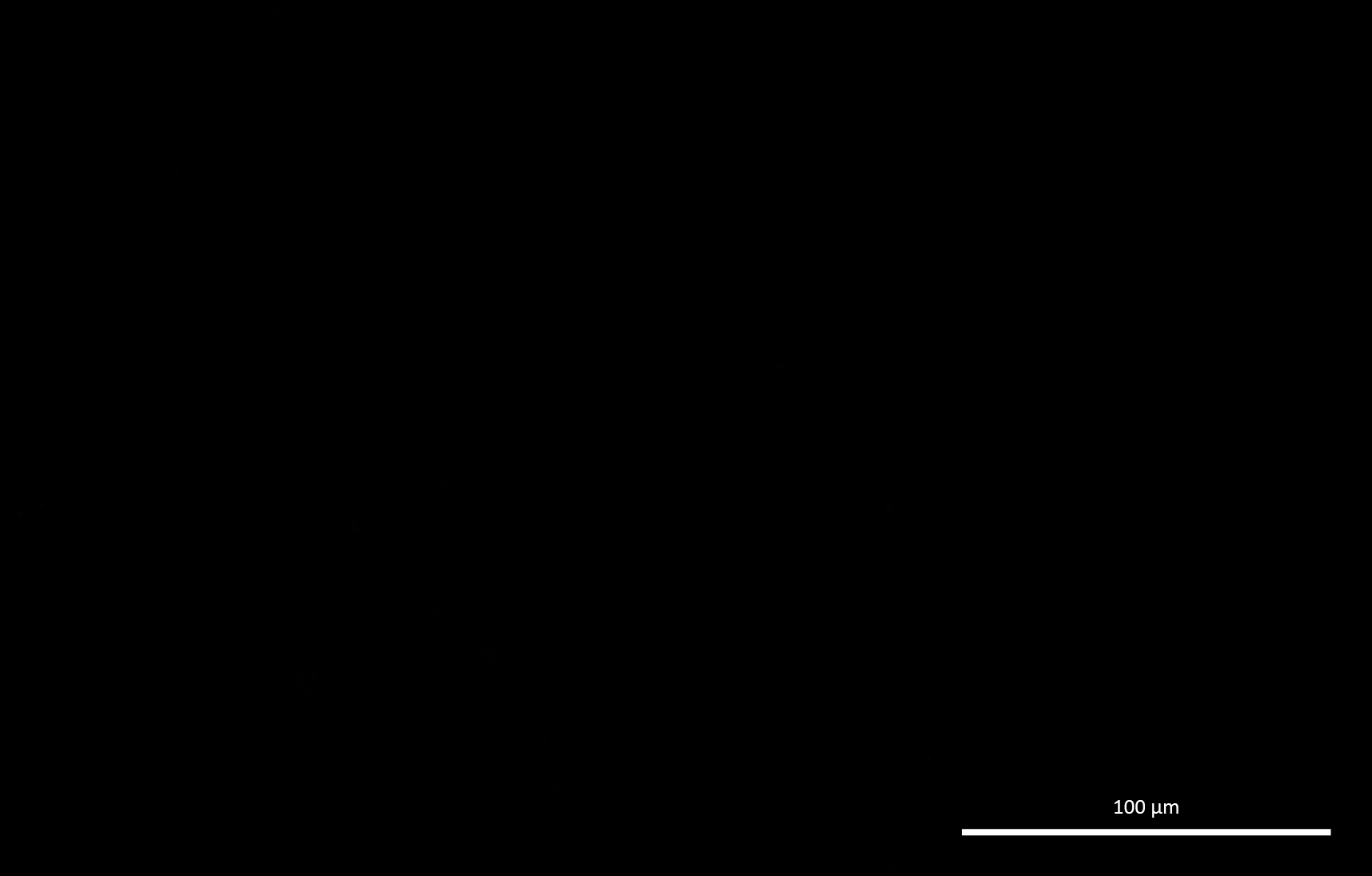

Supplement: Supplementary file 7 — Source Data Fig. 1 [file 44319_2023_24_MOESM7_ESM.zip › Figure 1/1B/HFFTH_D22_NegControl_-Image Export-04/HFFTH_D22_-Control_647-02-Image Export-04_Alexa Fluor 488_2.tif]

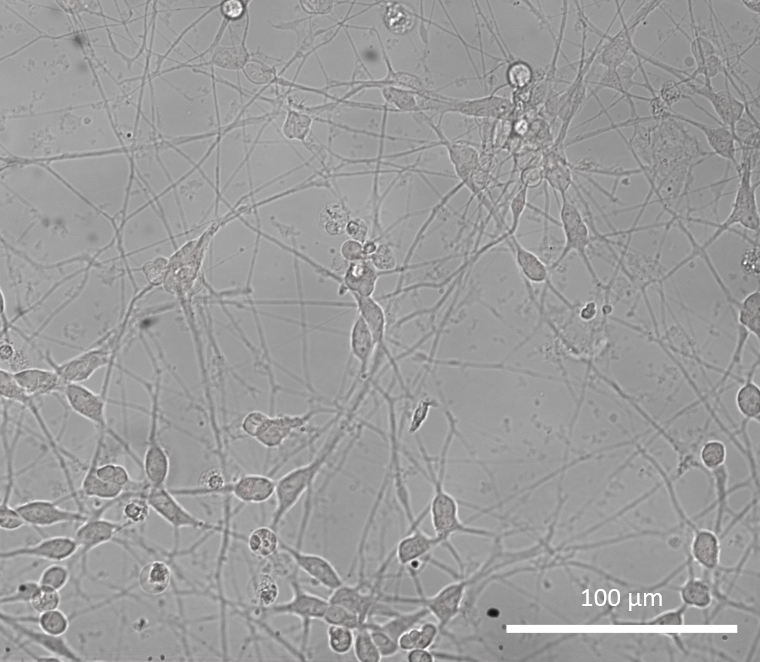

Supplement: Supplementary file 7 — Source Data Fig. 1 [file 44319_2023_24_MOESM7_ESM.zip › Figure 1/1B/HFFTHmCherry_LiveImaging_+sort-02-Image Export-06/HFFTHmCherry_LiveImaging_+sort-02-Image Export-06_c1-2.tif]

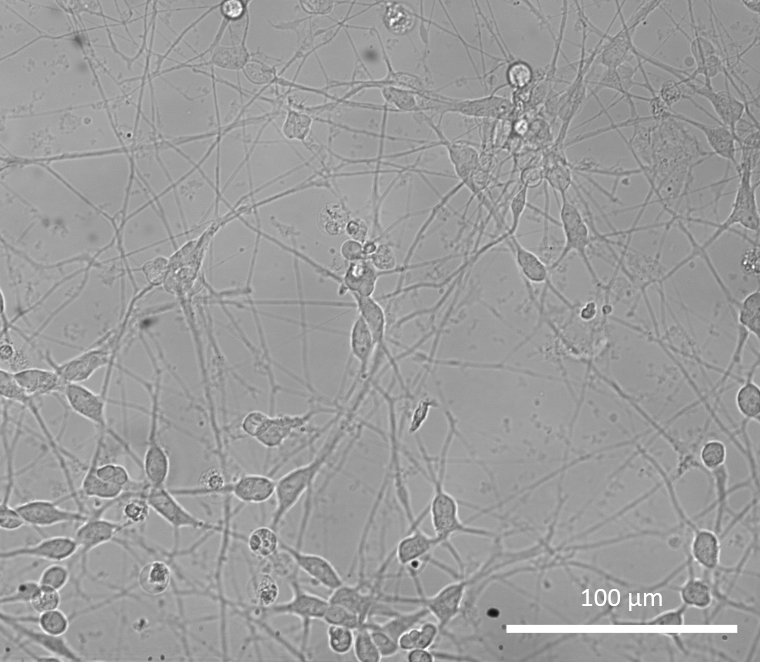

Supplement: Supplementary file 7 — Source Data Fig. 1 [file 44319_2023_24_MOESM7_ESM.zip › Figure 1/1B/HFFTHmCherry_LiveImaging_+sort-02-Image Export-06/HFFTHmCherry_LiveImaging_+sort-02-Image Export-06_TL Phase_1.tif]

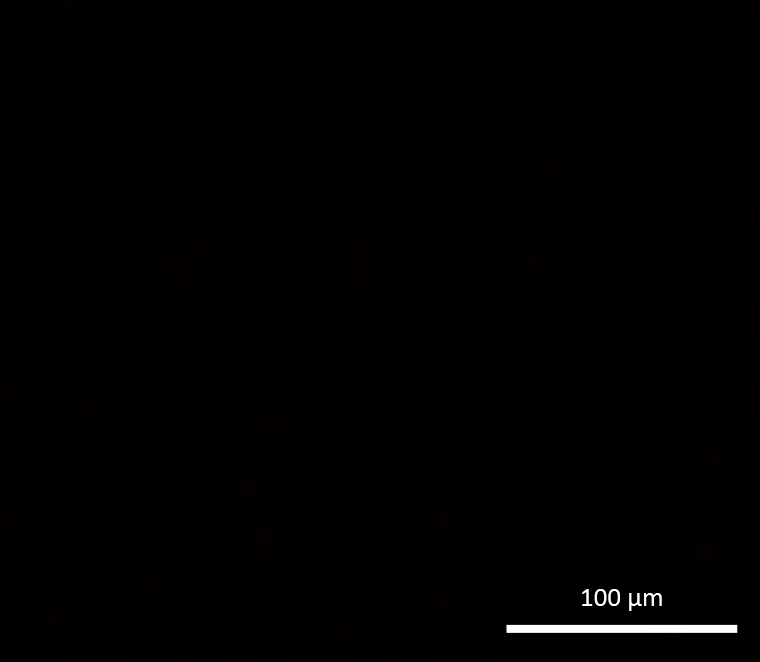

Supplement: Supplementary file 7 — Source Data Fig. 1 [file 44319_2023_24_MOESM7_ESM.zip › Figure 1/1B/HFFTHmCherry_LiveImaging_+sort-02-Image Export-06/HFFTHmCherry_LiveImaging_+sort-02-Image Export-06_mCherry_2.tif]

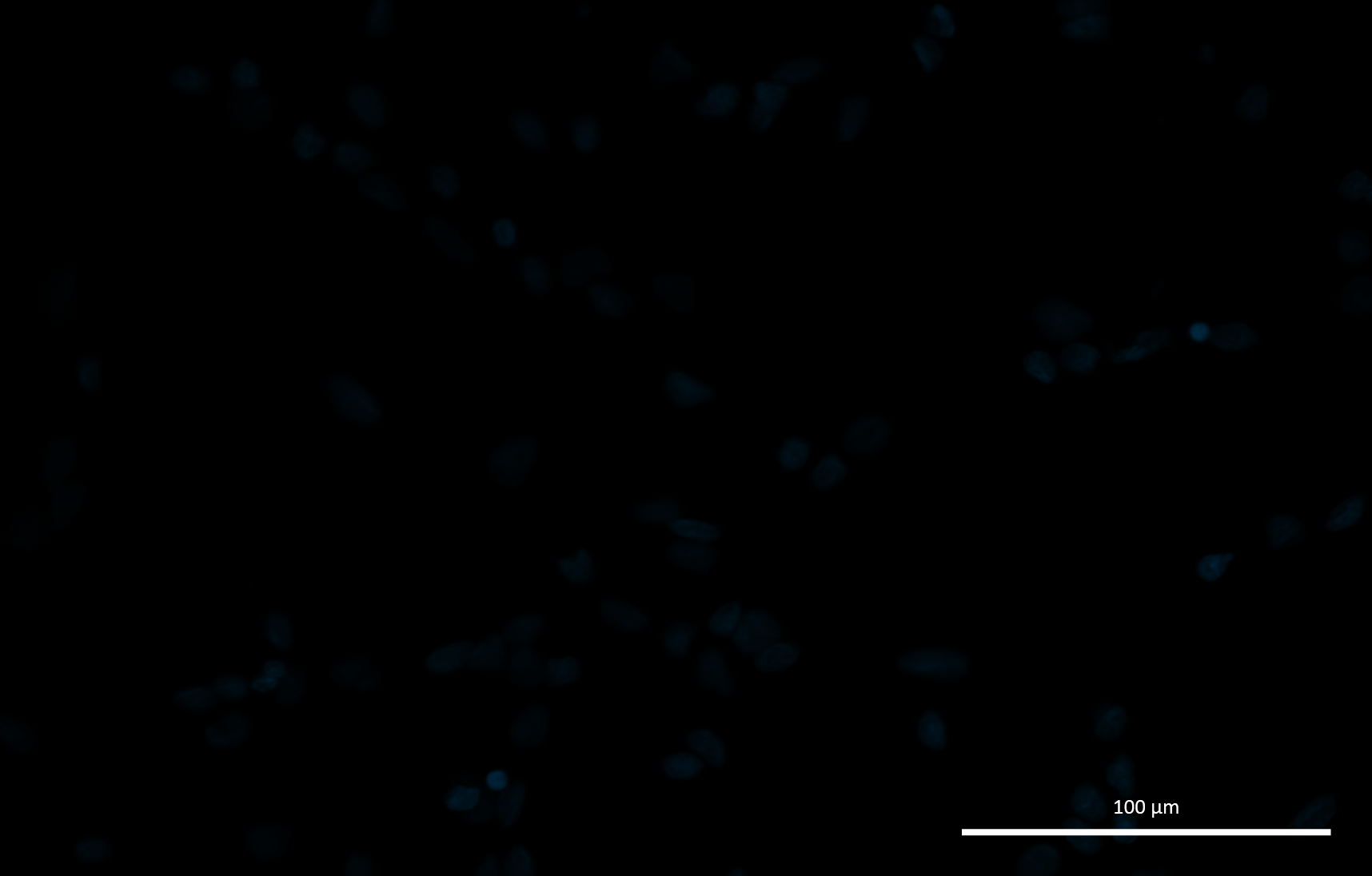

Supplement: Supplementary file 7 — Source Data Fig. 1 [file 44319_2023_24_MOESM7_ESM.zip › Figure 1/1B/HFFTH_D22_TH647_NeuN488_-sort-04-Image Export-14/HFFTH_D22_TH647_NeuN488_-sort-04-Image Export-14_DAPI_3.tif]

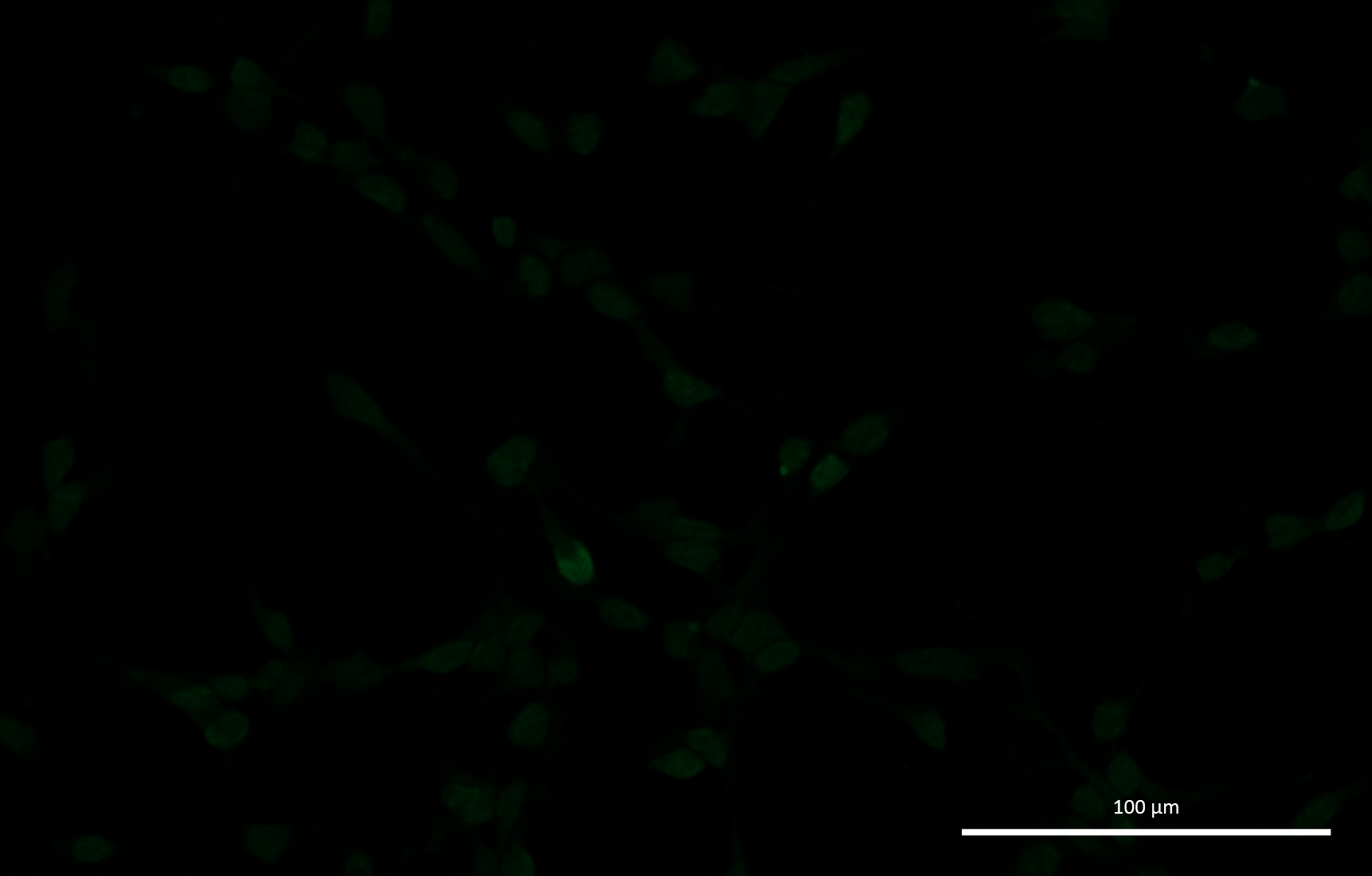

Supplement: Supplementary file 7 — Source Data Fig. 1 [file 44319_2023_24_MOESM7_ESM.zip › Figure 1/1B/HFFTH_D22_TH647_NeuN488_-sort-04-Image Export-14/HFFTH_D22_TH647_NeuN488_-sort-04-Image Export-14_Alexa Fluor 488_2.tif]

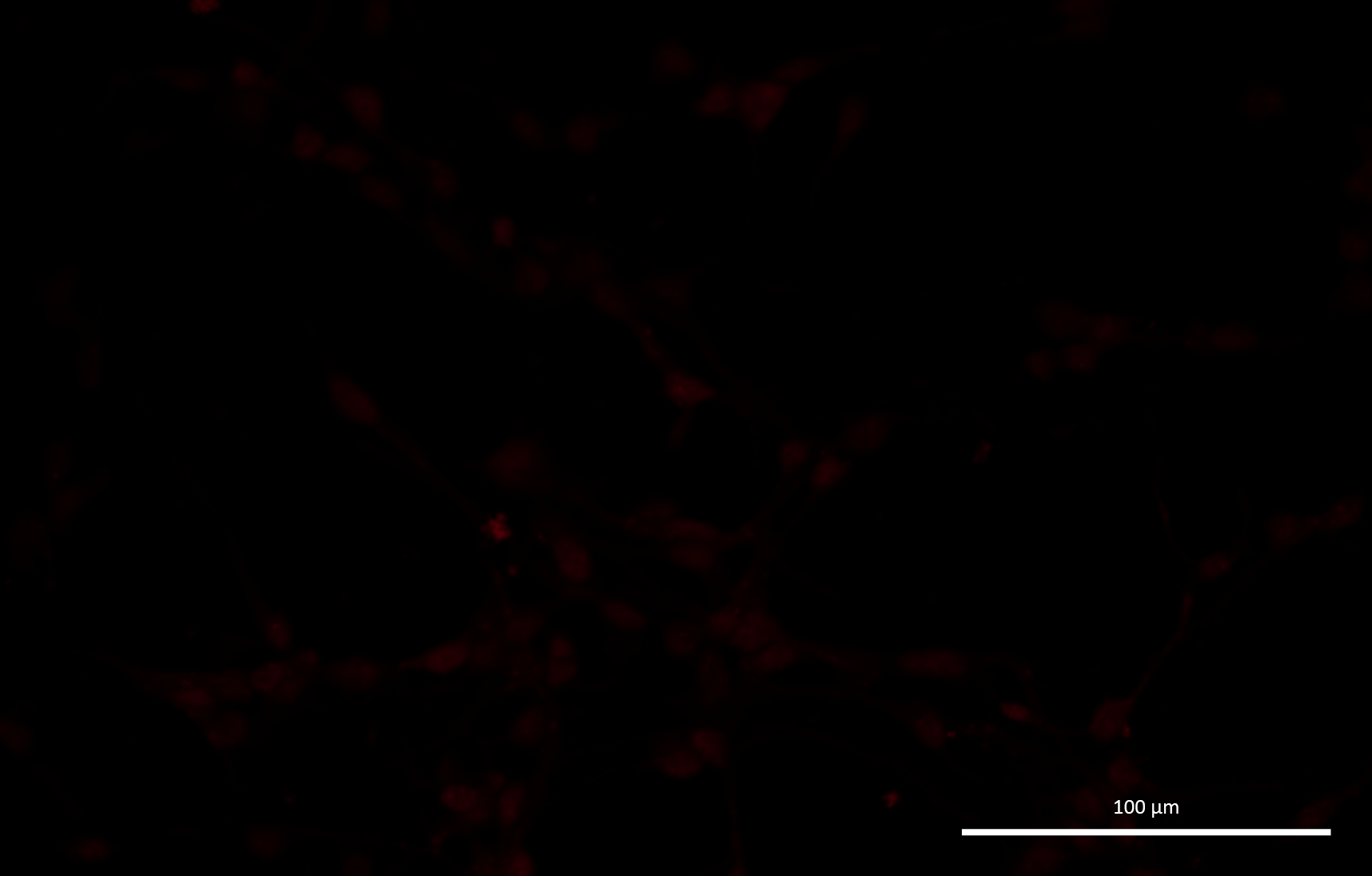

Supplement: Supplementary file 7 — Source Data Fig. 1 [file 44319_2023_24_MOESM7_ESM.zip › Figure 1/1B/HFFTH_D22_TH647_NeuN488_-sort-04-Image Export-14/HFFTH_D22_TH647_NeuN488_-sort-04-Image Export-14_Alexa Fluor 647_1.tif]

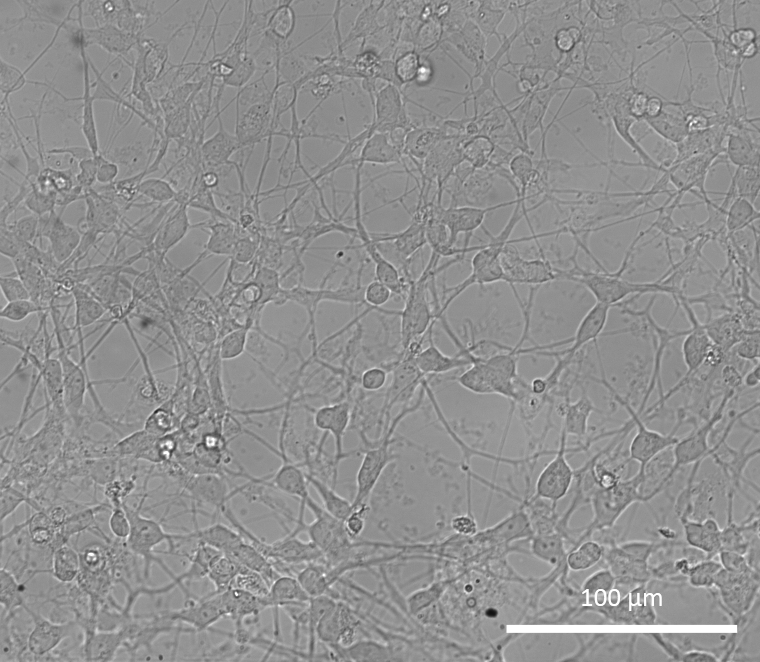

Supplement: Supplementary file 7 — Source Data Fig. 1 [file 44319_2023_24_MOESM7_ESM.zip › Figure 1/1B/HFFTHmCherry_LiveImaging_unsorted-04-Image Export-04/HFFTHmCherry_LiveImaging_unsorted-04-Image Export-04_TL Phase_1.tif]

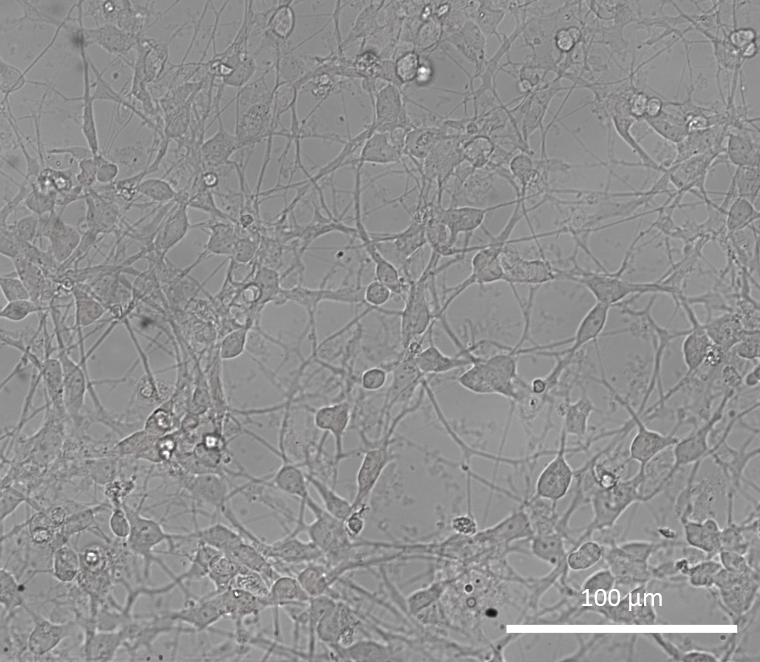

Supplement: Supplementary file 7 — Source Data Fig. 1 [file 44319_2023_24_MOESM7_ESM.zip › Figure 1/1B/HFFTHmCherry_LiveImaging_unsorted-04-Image Export-04/HFFTHmCherry_LiveImaging_unsorted-04-Image Export-04_c1-2.tif]

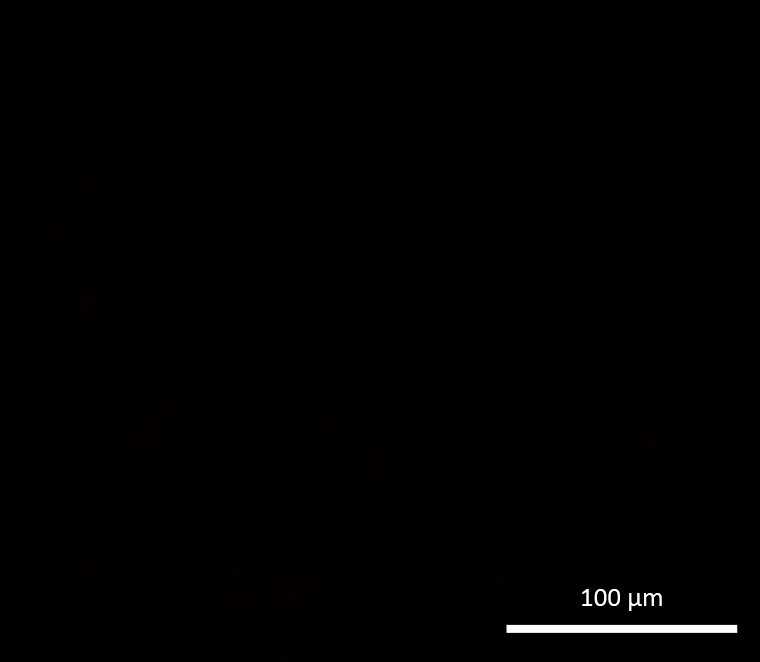

Supplement: Supplementary file 7 — Source Data Fig. 1 [file 44319_2023_24_MOESM7_ESM.zip › Figure 1/1B/HFFTHmCherry_LiveImaging_unsorted-04-Image Export-04/HFFTHmCherry_LiveImaging_unsorted-04-Image Export-04_mCherry_2.tif]

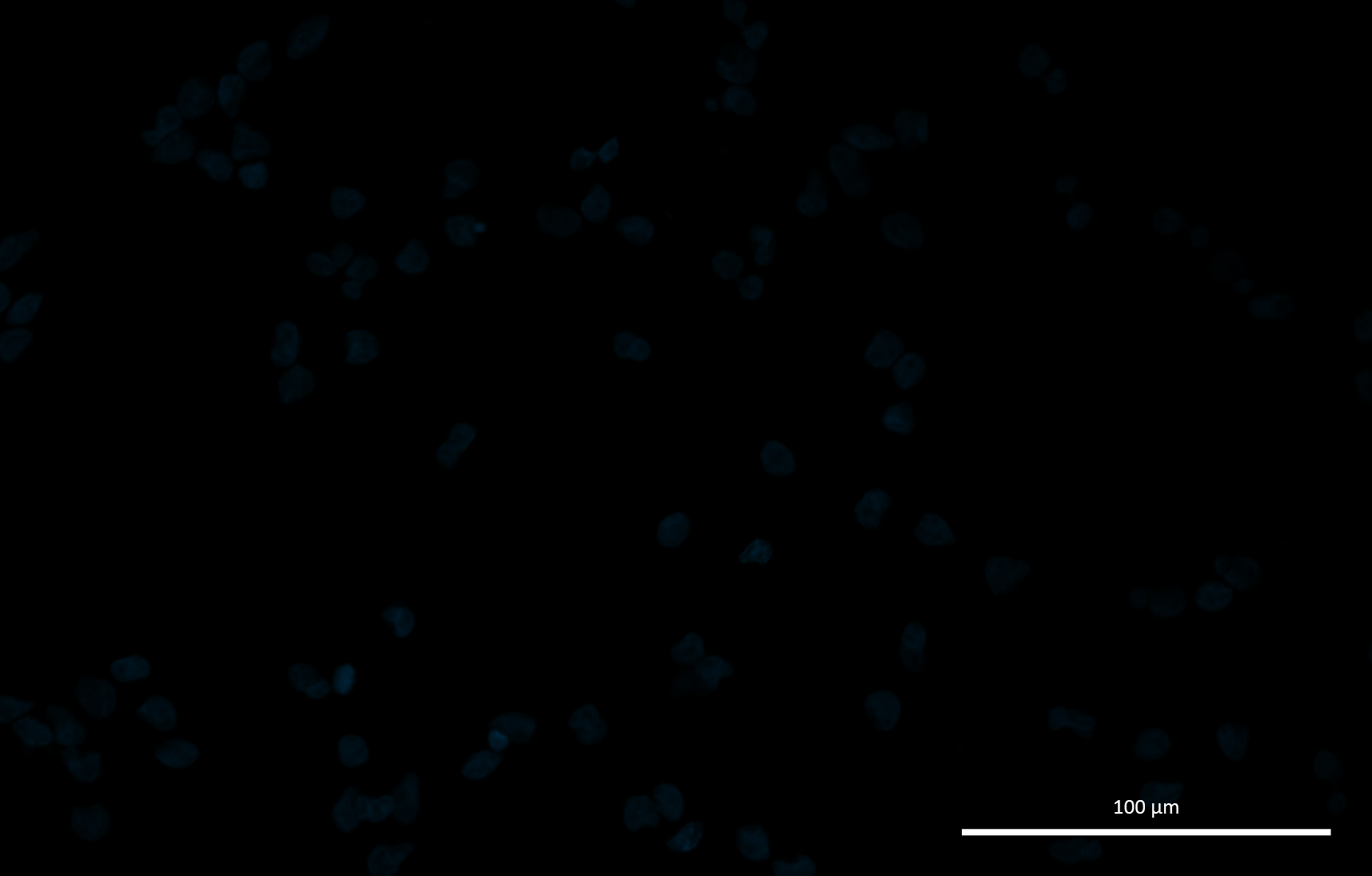

Supplement: Supplementary file 7 — Source Data Fig. 1 [file 44319_2023_24_MOESM7_ESM.zip › Figure 1/1B/HFFTH_D22_TH647_NeuN488_+sort-Image Export-07/HFFTH_D22_TH647_NeuN488_+sort-Image Export-07_DAPI_3.tif]

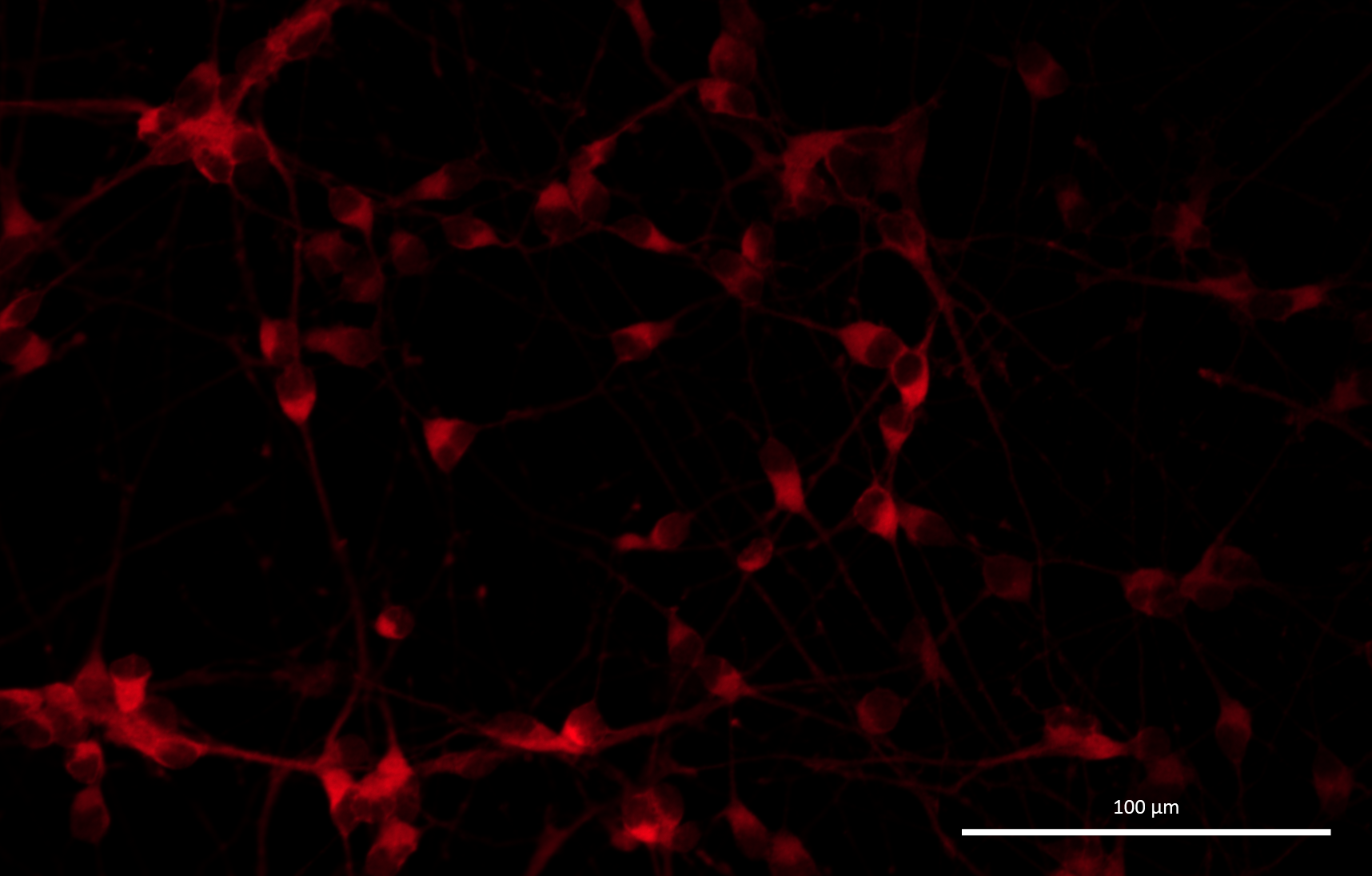

Supplement: Supplementary file 7 — Source Data Fig. 1 [file 44319_2023_24_MOESM7_ESM.zip › Figure 1/1B/HFFTH_D22_TH647_NeuN488_+sort-Image Export-07/HFFTH_D22_TH647_NeuN488_+sort-Image Export-07_Alexa Fluor 647_1.tif]

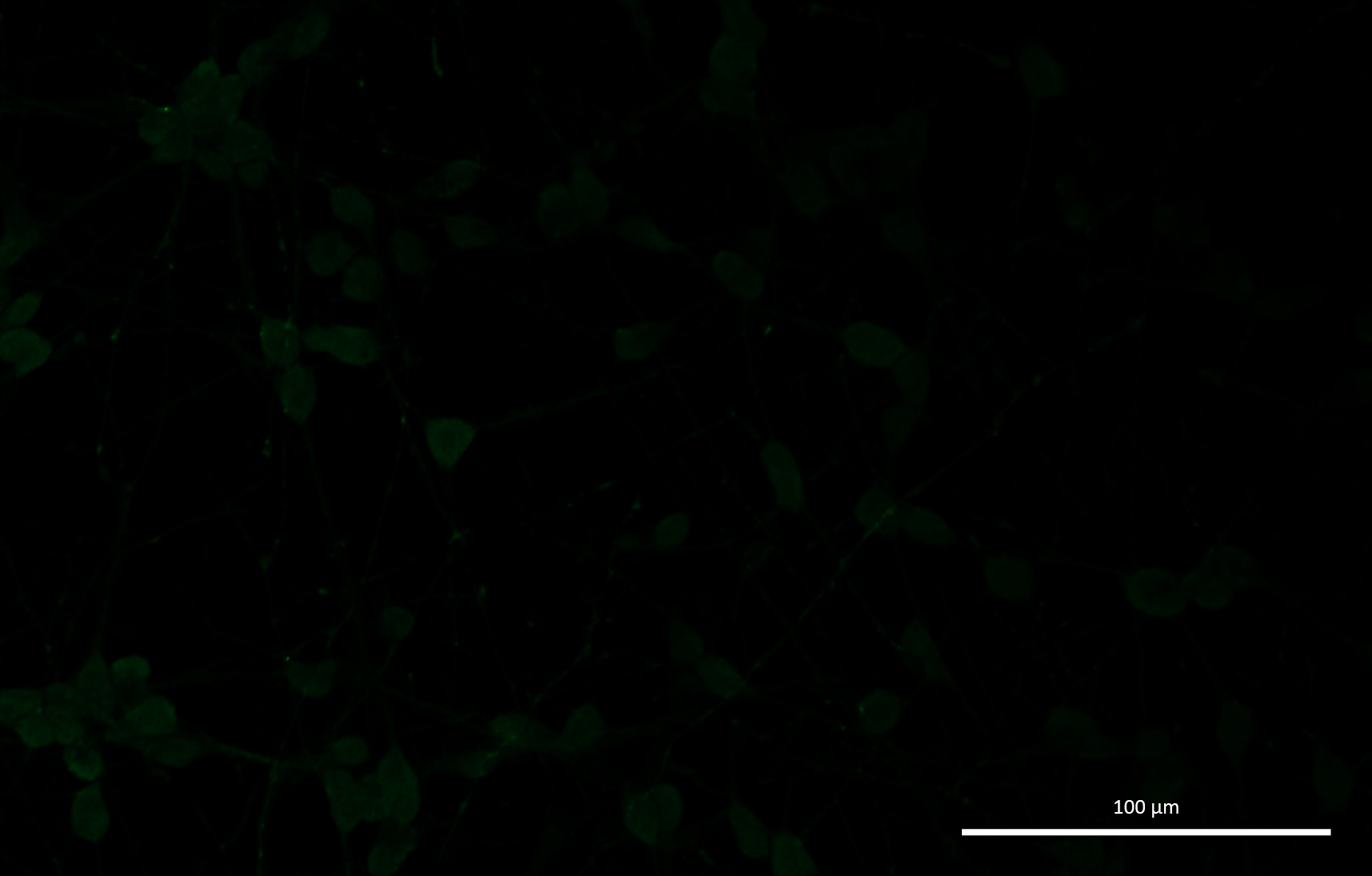

Supplement: Supplementary file 7 — Source Data Fig. 1 [file 44319_2023_24_MOESM7_ESM.zip › Figure 1/1B/HFFTH_D22_TH647_NeuN488_+sort-Image Export-07/HFFTH_D22_TH647_NeuN488_+sort-Image Export-07_Alexa Fluor 488_2.tif]

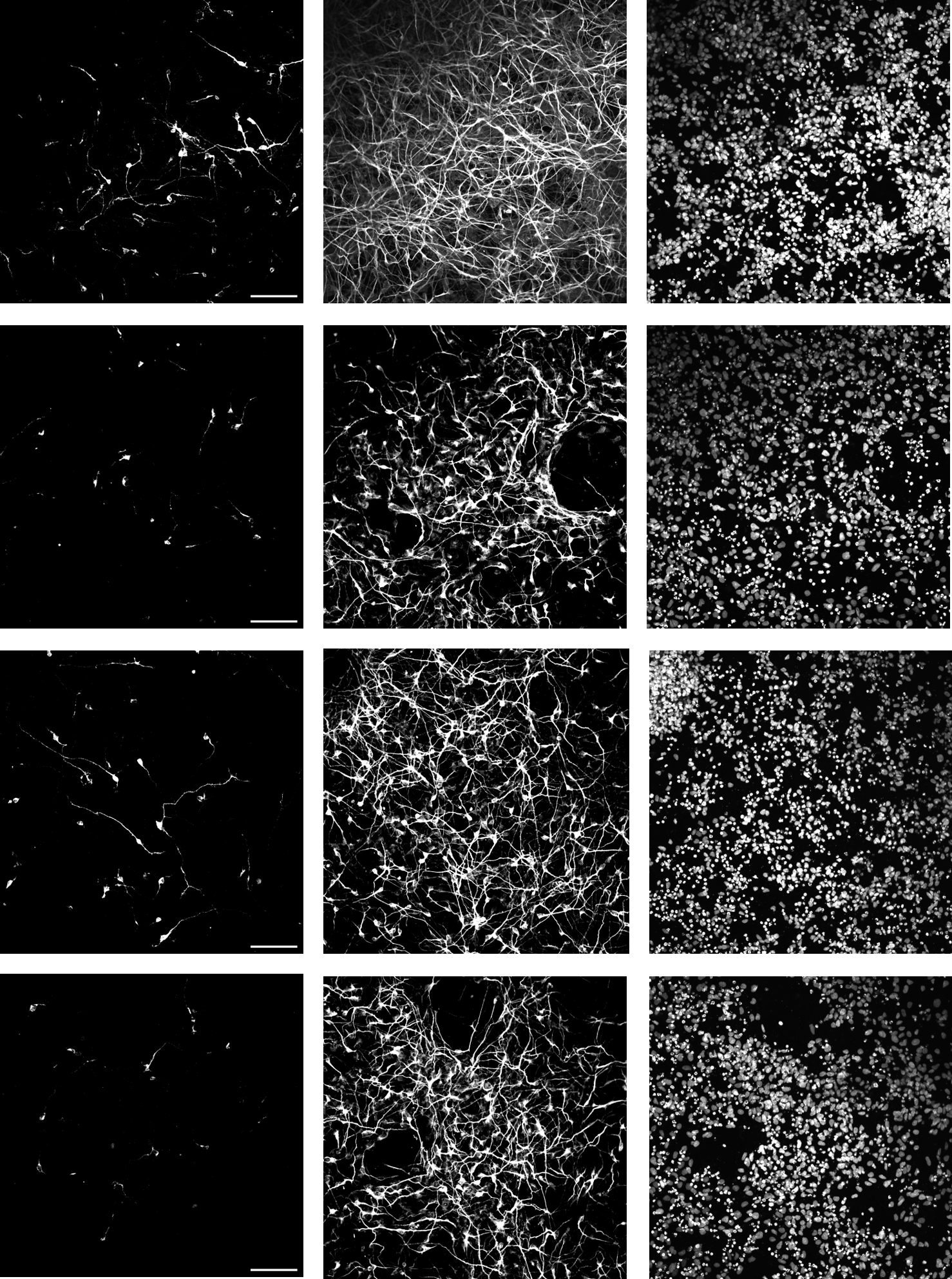

Supplement: Supplementary file 8 — Source Data Fig. 4 [file 44319_2023_24_MOESM8_ESM.zip › Figure 4/4D/17608.tif]

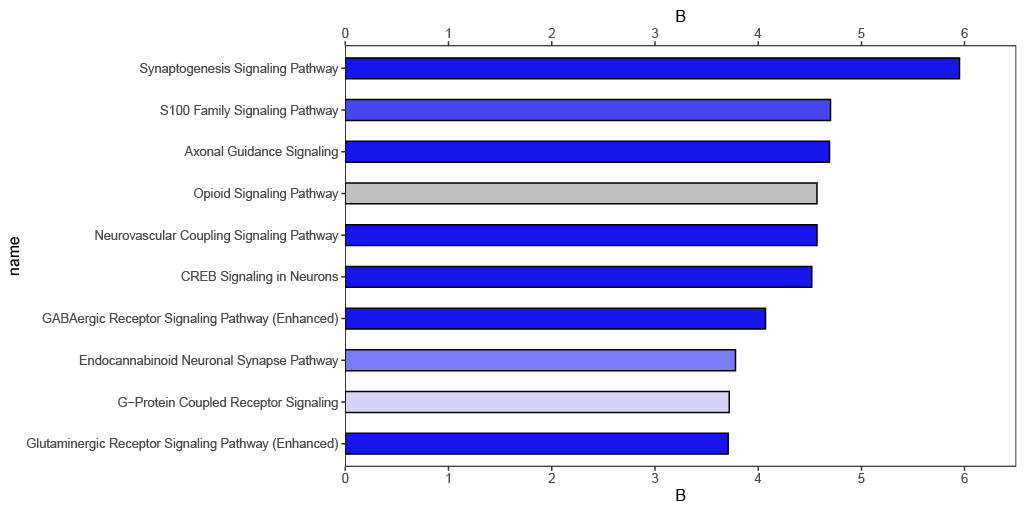

Supplement: Supplementary file 11 — Source Data Fig. 7 [file 44319_2023_24_MOESM11_ESM.zip › Figure 7/7B/IPA_results.tiff]
