# Supplementary material for: Multiomics analysis identifies novel facilitators of human dopaminergic neuron differentiation
Source: EMBO Rep. 2023 Dec 19;25(1):17. doi: 10.1038/s44319-023-00024-2 (PMC10897179; doi:10.1038/s44319-023-00024-2)
Supplement: Supplementary file 13 — Expanded View Figures [file 44319_2023_24_MOESM13_ESM.pdf]

# Expanded View Figures

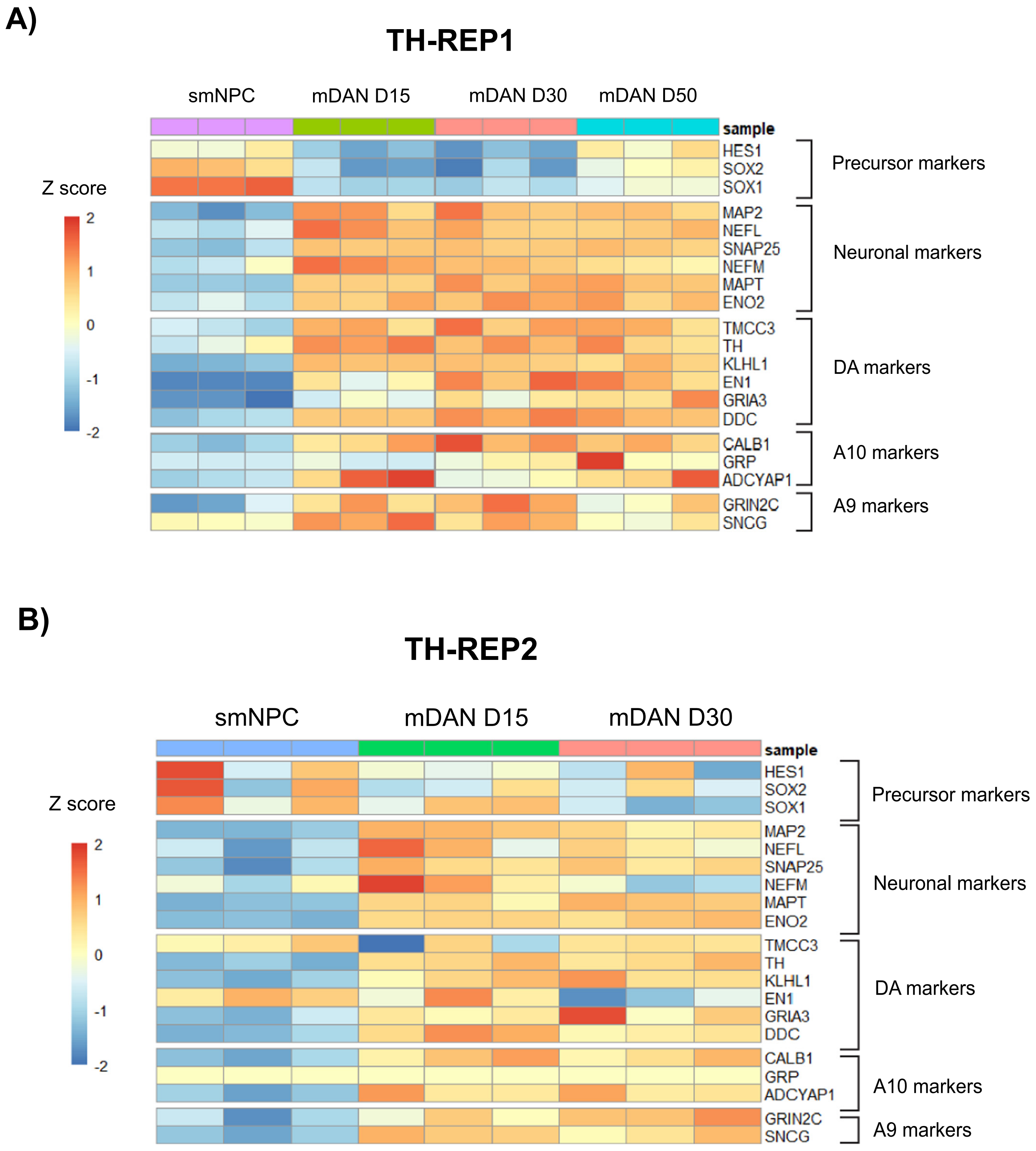


**Figure EV1. Expression of cell type-specific marker genes during mDAN differentiation.**

(**A**) Heatmap showing the expression of cell-type-specific markers of smNPCs, neurons, mDANs, and mDAN subtypes selected from literature (Anderegg et al, 2015; La Manno et al, 2016) in TH-Rep1 cell line. (**B**) Heatmap showing the expression of cell-type-specific markers from panel A in TH-Rep2 cell line. GRP A10 marker gene was not expressed in this iPSC cell line.

 © The Author(s)

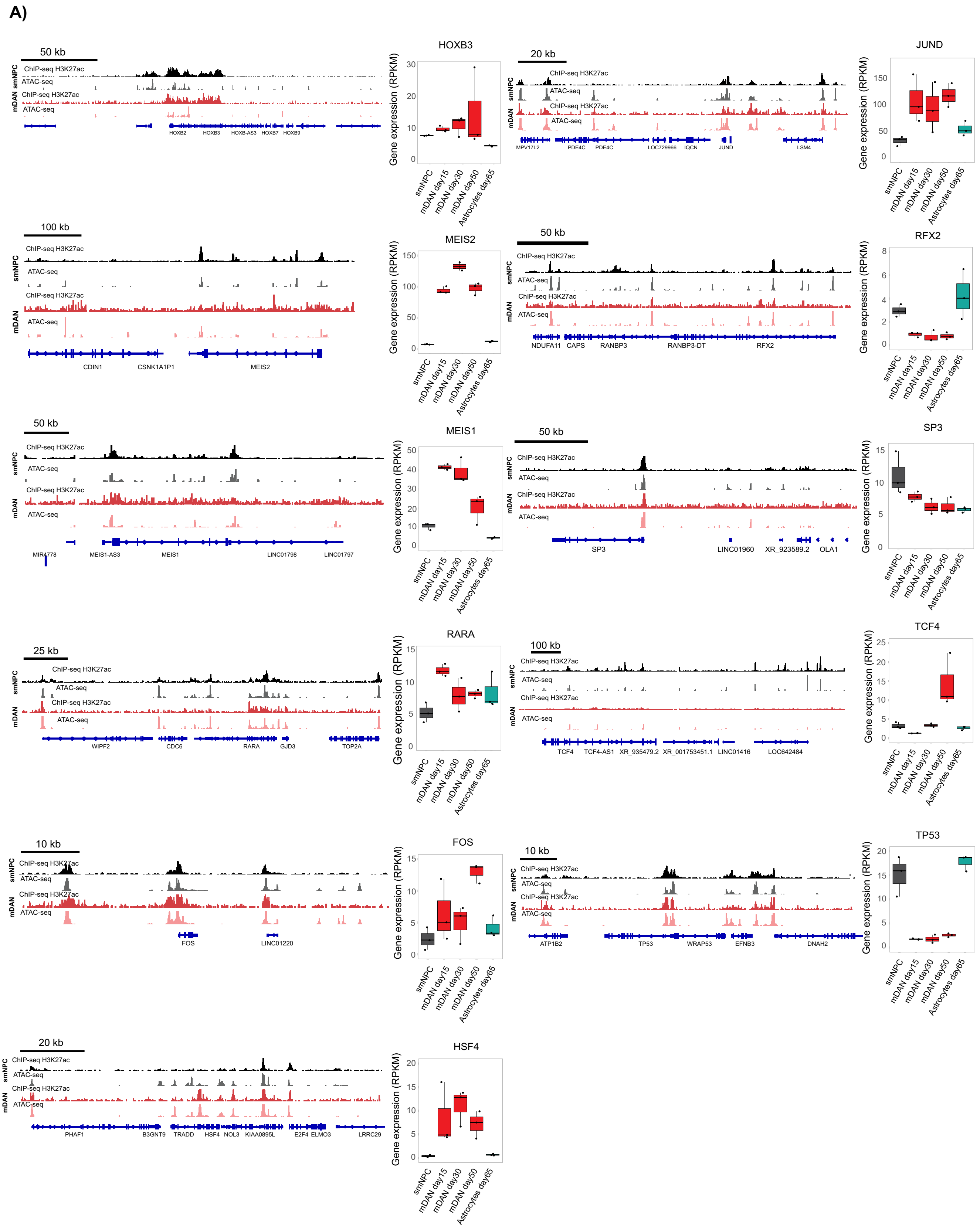
A)
HOXB3
JUND
MEIS2
RFX2
MEIS1
SP3
RARA
TCF4
FOS
TP53
HSF4
Gene expression (RPKM)
smNPC
mDAN day15
mDAN day30
mDAN day50
Astrocytes day65
ChIP-seq H3K27ac
ATAC-seq
smNPC
mDAN
50 kb
20 kb
100 kb
25 kb
10 kb
HOXB2 HOXB3 HOXB-AS3 HOXB7 HOXB9
MPV17L2 PDE4C PDE4C LOC729966 IQCN JUND LSM4
CDIN1 CSNK1A1P1 MEIS2
NDUFA11 CAPS RANBP3 RANBP3-DT RFX2
MIR4778 MEIS1-AS3 MEIS1 LINC01798 LINC01797
SP3 LINC01960 XR_923589.2 OLA1
WIPF2 CDC6 RARA GJD3 TOP2A
TCF4 TCF4-AS1 XR_935479.2 XR_001753451.1 LINC01416 LOC642484
FOS LINC01220
ATP1B2 TP53 WRAP53 EFNB3 DNAH2
PHAF1 B3GNT9 TRADD HSF4 NOL3 KIAA0895L E2F4 ELMO3 LRRC29

**Figure EV2. TFs controlled by super-enhancers in mDANs.**

H3K27ac signal and chromatin accessibility profiles together with the expression dynamics during mDAN differentiation of the 11 additional TFs under the control of SEs between day 30 and day 50 and predicted by EPIC-DREM. ATAC-seq and ChIP-seq tracks are plotted under the same scale per dataset for comparison purposes. Data are representative of 3 independent experiments. Boxplots illustrate the distribution of data as described in Fig. 1.


© The Author(s)

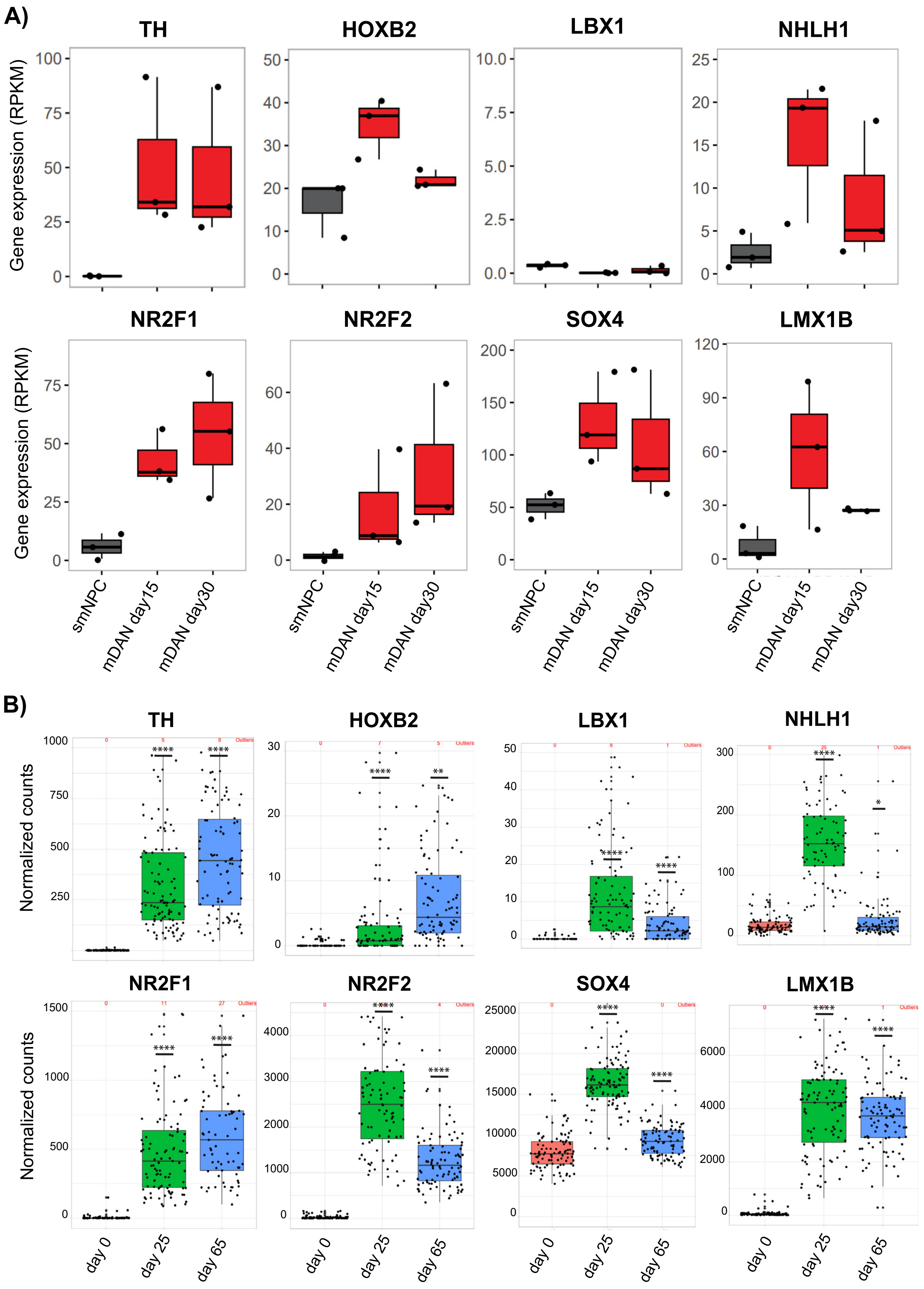
A)
TH
HOXB2
LBX1
NHLH1
NR2F1
NR2F2
SOX4
LMX1B
Gene expression (RPKM)
smNPC
mDAN day15
mDAN day30
B)
Normalized counts
day 0
day 25
day 65
Outliers

**Figure EV3. Expression dynamics of candidate TFs in mDAN differentiation across different cell lines.**

(**A**) Expression dynamics of the novel candidate TFs during mDAN differentiation of the TH-Rep2 cell line. (**B**) Expression dynamics of the novel candidate TFs in 95 independent iPSC-lines during mDANs differentiation. The RNA-seq data was kindly provided by the Foundational Data Initiative for Parkinson's Disease (FOUNDIN-PD) consortium (Bressan et al, 2023). Data information: Data are representative of 3 (**A**) and 95 (**B**) independent experiments. Two samples *t* test was used for statistical analysis (**B**) with day 0 used as the reference sample. *$p$ value <0.05, **$p$ value <0.01, ****$p$ value <0.0001, and ns not significant. Boxplots illustrate the distribution of data as described in Fig. 1.

 © The Author(s)

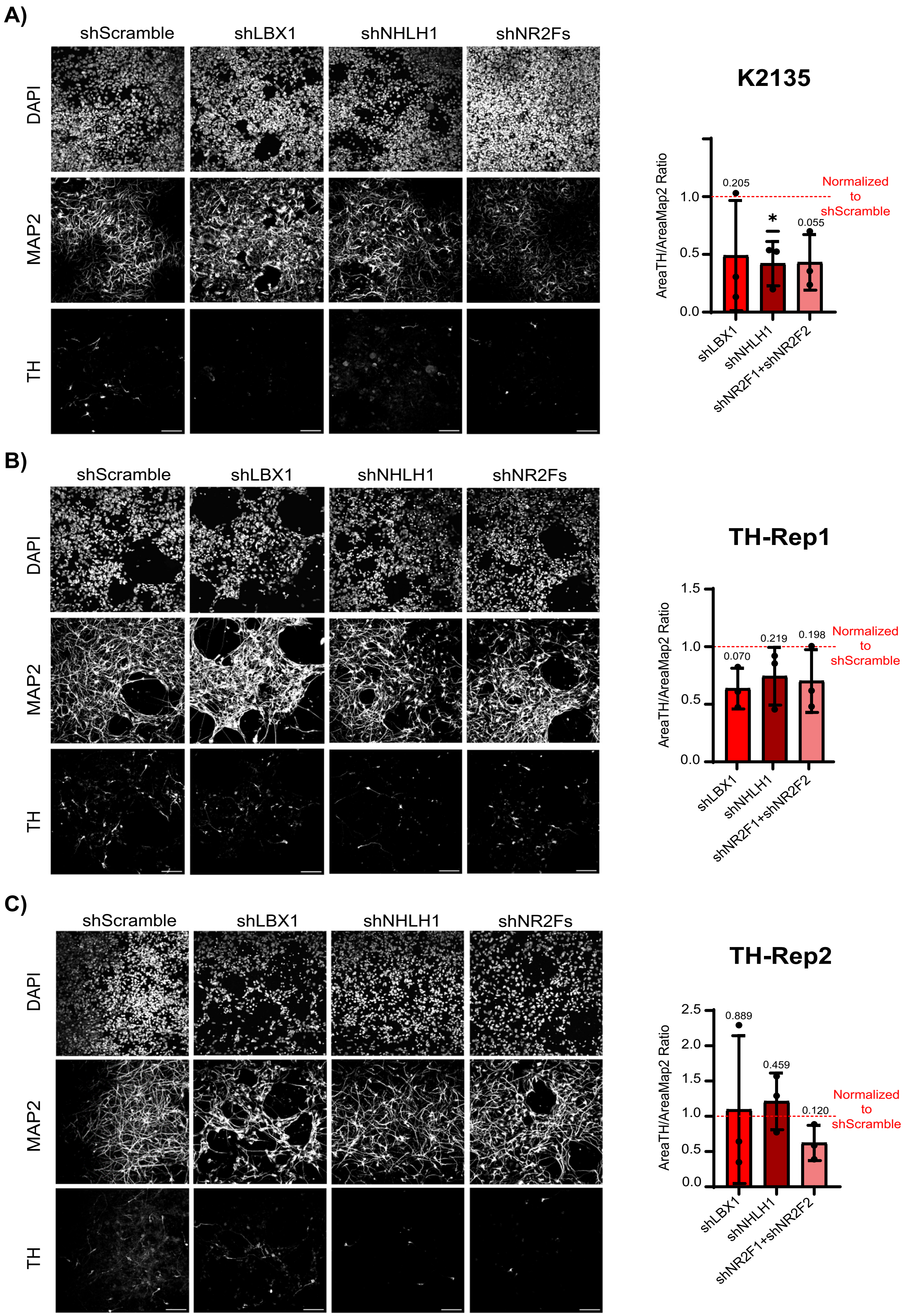
A)
shScramble
shLBX1
shNHLH1
shNR2Fs
DAPI
MAP2
TH
K2135
AreaTH/AreaMap2 Ratio
0.205
*
0.055
Normalized to shScramble
shLBX1
shNHLH1
shNR2F1+shNR2F2
B)
shScramble
shLBX1
shNHLH1
shNR2Fs
DAPI
MAP2
TH
TH-Rep1
AreaTH/AreaMap2 Ratio
0.070
0.219
0.198
Normalized to shScramble
shLBX1
shNHLH1
shNR2F1+shNR2F2
C)
shScramble
shLBX1
shNHLH1
shNR2Fs
DAPI
MAP2
TH
TH-Rep2
AreaTH/AreaMap2 Ratio
0.889
0.459
0.120
Normalized to shScramble
shLBX1
shNHLH1
shNR2F1+shNR2F2

**Figure EV4. NHLH1, LBX1, NR2F1/2 are necessary for mDAN differentiation in multiple cell lines.**

(**A–C**) High-content imaging analysis of (**A**) K2135 cell line, (**B**) TH-Rep1 cell line, and (**C**) TH-Rep2 cell line at day 15 of differentiation following late transduction with shRNA lentiviral particles. Cells were stained for the nuclear marker DAPI, the neuronal marker MAP2 and the marker for mDANs TH. Scale bar = 100 µm. Quantification of the TH-stained area over the MAP2 area. Ratios were normalized to the shSCRAMBLE per replicate. Data are representative of 3 independent experiments. Error bars correspond to ±1 standard deviation (SD) from the mean. One sample *t* test was used for statistical analysis, taking 1 as the theoretical mean for TH quantification. *$p$ value <0.05.


© The Author(s)
